# Hierarchical Incorporation of Reduced Graphene Oxide into Anisotropic Cellulose Nanofiber Foams Improves Their Thermal Insulation

**DOI:** 10.1021/acsami.4c09654

**Published:** 2024-08-13

**Authors:** Seyed Ehsan Hadi, Elias Möller, Sina Nolte, Agnes Åhl, Olivier Donzel-Gargand, Lennart Bergström, Alexander Holm

**Affiliations:** † Department of Materials and Environmental Chemistry, Stockholm University, 106 91 Stockholm, Sweden; ‡ Wallenberg Wood Science Center, Department of Materials and Environmental Chemistry, Stockholm University, 10691 Stockholm, Sweden; § Department of Chemistry, Philipps-Universität Marburg, 35032 Marburg, Germany; ∥ Institute of Inorganic Chemistry, Leibniz University Hannover, D-30167 Hannover, Germany; ⊥ Ångström Solar Center, Division of Solar Cell Technology, Uppsala University, 751 21 Uppsala, Sweden; # Laboratory of Organic Electronics, Department of Science and Technology (ITN), Linköping University, SE-60174 Norrköping, Sweden

**Keywords:** cellulose nanofiber foam, thermal conductivity, reduced graphene oxide, layer-by-layer, self-assembly, insulation, CNF

## Abstract

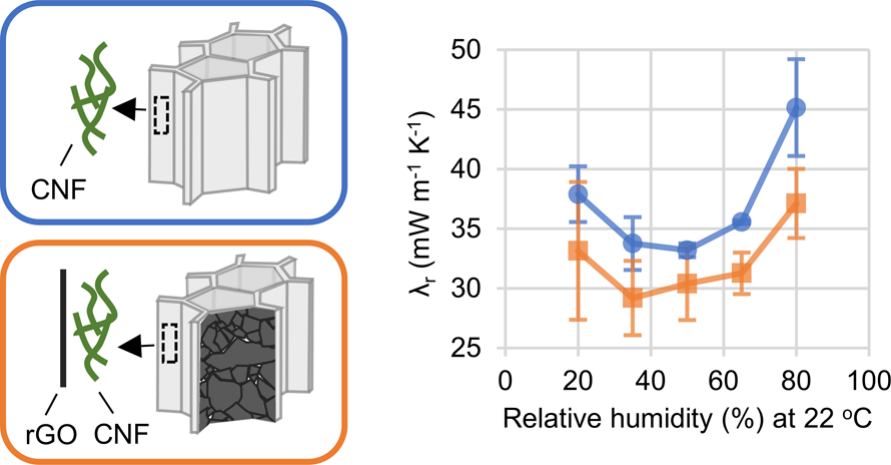

Anisotropic cellulose nanofiber (CNF) foams represent the state-of-the-art in renewable insulation. These foams consist of large (diameter >10 μm) uniaxially aligned macropores with mesoporous pore-walls and aligned CNF. The foams show anisotropic thermal conduction, where heat transports more efficiently in the axial direction (along the aligned CNF and macropores) than in the radial direction (perpendicular to the aligned CNF and macropores). Here we explore the impact on axial and radial thermal conductivity upon depositing a thin film of reduced graphene oxide (rGO) on the macropore walls in anisotropic CNF foams. To obtain rGO films on the foam walls we developed liquid-phase self-assembly to deposit rGO in a layer-by-layer fashion. Using electron and ion microscopy, we thoroughly characterized the resulting rGO-CNF foams and confirmed the successful deposition of rGO. These hierarchical rGO-CNF foams show lower radial thermal conductivity (λ_r_) across a wide range of relative humidity compared to CNF control foams. Our work therefore demonstrates a potential method for improved thermal insulation in anisotropic CNF foams and introduces versatile self-assembly for postmodification of such foams.

## Introduction

Ten percent of world energy consumption is used for heating or cooling buildings.^1^ Improving the insulation capacity of insulation materials could therefore substantially reduce global energy need.^2,3^ Recent advances in the field of thermal insulation have focused on the development of lightweight composite aerogels/foams.^4−8^ Anisotropic cellulose nanofiber (CNF) foams and aerogels are particularly interesting in this context, showing thermal conductivities^9,10^ that are lower than, or similar to commercial materials, such as expanded polystyrene (λ ≈ 30–40 mW m^–1^ K^–1^), polyurethane (λ ≈ 20–30 mW m^–1^ K^–1^), and mineral wool (λ ≈ 30–40 mW m^–1^ K^–1^).^11^ Because of their excellent insulation capacity, there has been increasing interest in further developing anisotropic CNF foams. As examples, there are efforts to improve their fire-retardancy,^10,12−14^ and mechanical properties,^10,14−16^ and to reduce their moisture sensitivity.^17−19^

**Figure 1:**
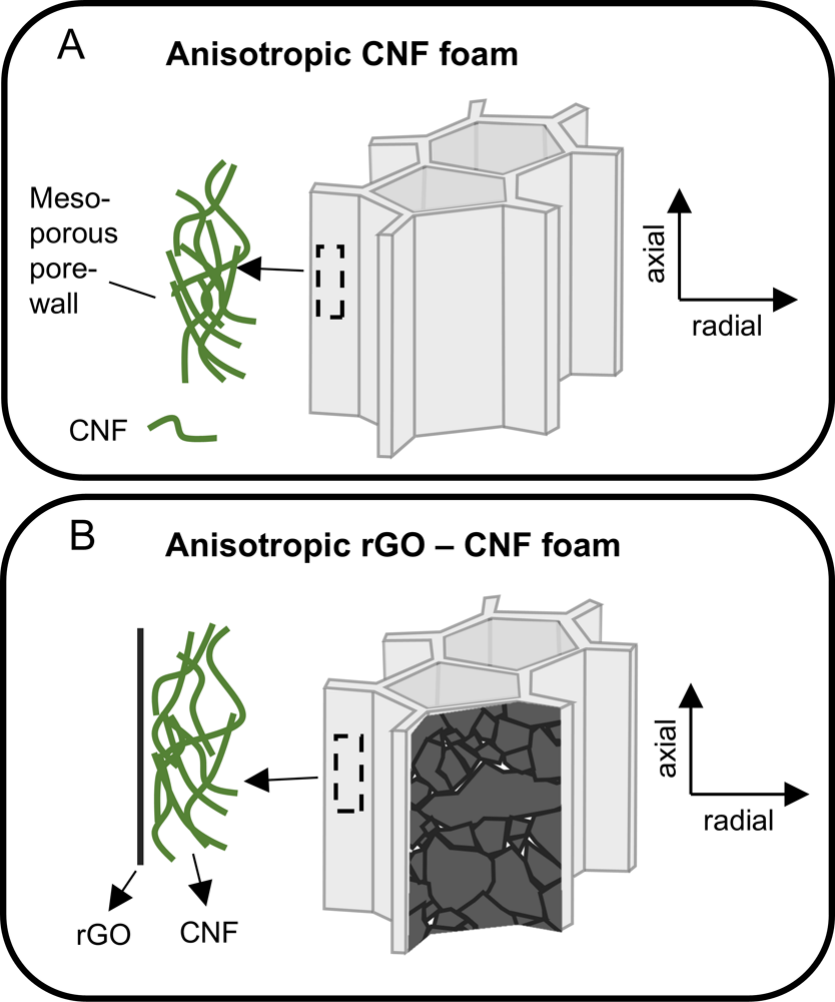
(A) Anisotropic, unidirectionally freeze-cast CNF foams. The foams consist of macropores (≈10–200 μm diameter, >1 mm long) extending in the axial direction, with mesoporous pore walls (≈3–15 nm pore diameter). (B). Illustration of the rGO-CNF foams produced in this work.

Anisotropic, unidirectionally freeze-cast CNF foams typically consist of large (≈10–200 μm diameter) elongated (>1 mm) macropores in the axial direction, with macropore walls being mesoporous (≈3–15 nm pore diameter).^9,10,20−22^ Furthermore, in the macropore walls, CNF is aligned in the axial direction (Figure 1A).^10,22,23^ The radial thermal conductivity (λ_r_) in these foams is significantly lower than the axial thermal conductivity (λ_a_) along the aligned nanofibers. Part of this thermal conduction anisotropy stems from reduced gas-conduction in the mesoporous walls.^10^ Another contribution stems from anisotropic solid conduction in individual CNF fibers. CNF is made up of aligned bundles of crystalline cellulose chains, interspersed with amorphous regions.^24^ The anisotropic thermal conduction in CNF is due to anisotropic conduction in these crystalline bundles, where numerical modeling suggests conduction along the crystalline sections is ≈2–8 times higher than across.^25,26^ Moreover, because of the large aspect ratios (*L*_axial_/*L*_radial_ > 100) of the crystalline sections in CNF,^9,27^ the phonon mean-free path is longer along than across the crystals, further augmenting thermal conduction anisotropy.^9,28^

The structural features of anisotropic CNF foams and the resulting low λ_r_ suggests that introduction of an additional highly anisotropic thermal conductor, aligned in the axial direction, could modulate the thermal conductivity. Such modulation is interesting not only for insulation applications, but also for directional thermal management of materials and devices.^29−32^ Reduced graphene oxide (rGO) is a highly anisotropic thermal conductor, and thin rGO films have shown in-plane thermal conductivity of 61,000 mW m^–1^ K^–1^ with cross-plane conductivity of only 90 mW m^–1^ K^–1^ (i.e., thermal conduction anisotropy of λ_∥_/λ_⊥_ = 675).^33^ In addition, adding rGO along the plane of CNF paper has been shown to reduce the cross-plane thermal conductivity, while significantly increasing the in-plane thermal conduction.^34^ We therefore wanted to explore how deposition of a thin rGO film on the macropore walls of anisotropic CNF foams (Figure 1B) would change thermal conductivity of the foams.

In this work, we demonstrate a versatile layer-by-layer (LbL) method for hierarchical postmodification of anisotropic CNF foams. We also demonstrate that hierarchical incorporation of rGO is a potential strategy for improved thermal insulation in anisotropic CNF foams.

## Results and Discussion

### Self-Assembly of rGO onto the Macropore Walls of Anisotropic CNF Foams

**Figure 2:**
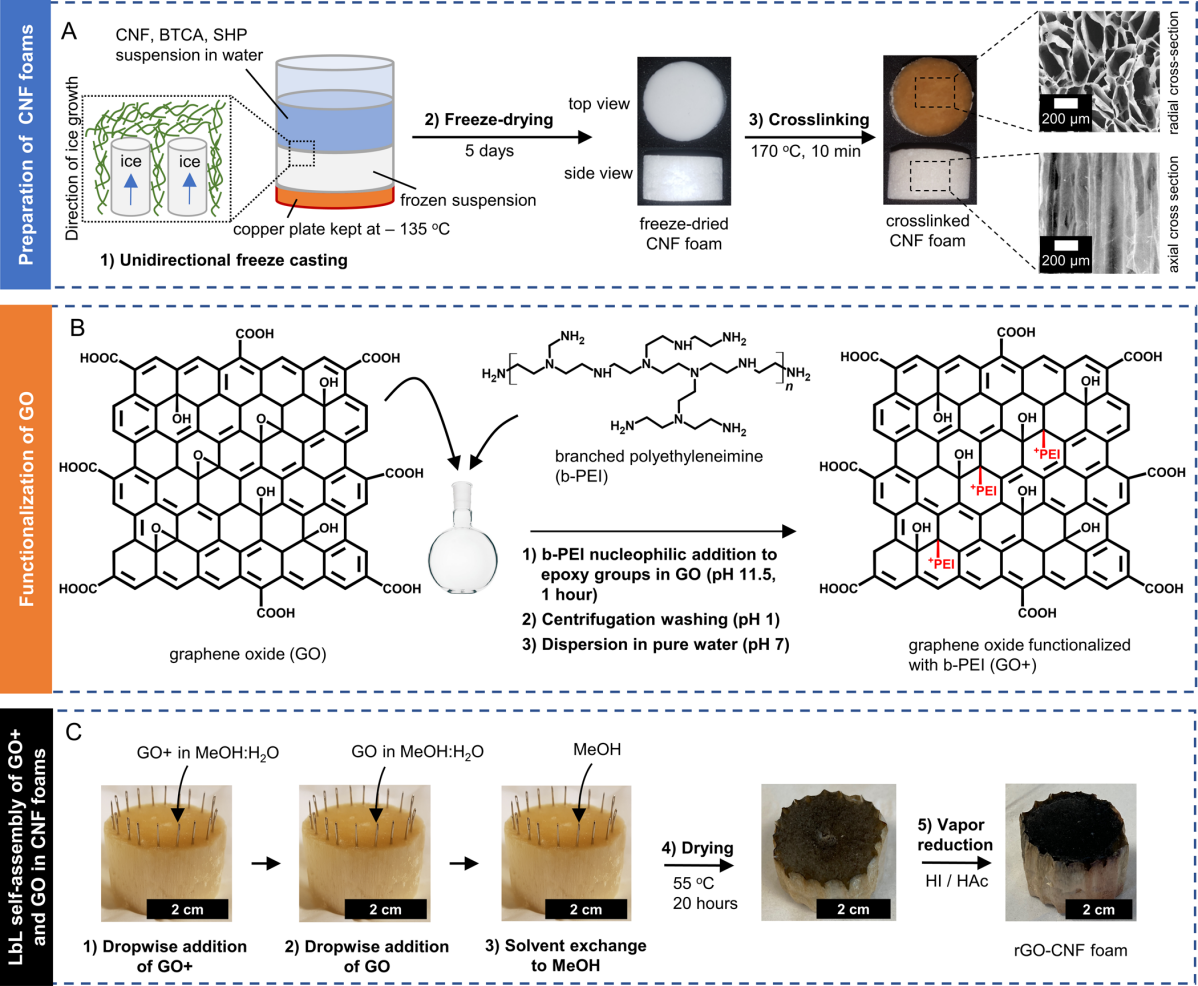
Production of hierarchical rGO-CNF foams. (A) Preparation of cross-linked CNF foams including (1) unidirectional freeze-casting (2) freeze-drying, and (3) cross-linking. (B) Functionalization of GO to make GO + by (1) branched polyethylene imine (b-PEI) nucleophilic addition to epoxy-groups in GO followed by (2) centrifugation washing, and (3) dispersion in pure water. (C) LbL self-assembly of GO+ and GO in anisotropic CNF foams, followed by vapor reduction, to make hierarchical rGO-CNF foams. Refer to the Experimental section for a detailed description of the synthesis of rGO-CNF foams.

CNF fibers were prepared by established protocols,^35,36^ using TEMPO oxidation. The resulting fibers had a charge of 0.56 mmol g^–1^ (from carboxylic acid residues), diameters of ≈1–3 nm, and lengths of ≈100–2000 nm (Figure S1), comparable to previous reports.^9,35^ CNF suspensions were then unidirectionally freeze-cast (Figure 2A) to produce anisotropic CNF foams, as previously described.^9,10^ To make CNF foams water-resilient, cross-linking is required,^18,37^ and we used butane tetracarboxylic acid (BTCA) as a cross-linker, as has previously been done with isotropic CNF foams.^37^ SEM characterization of our foams confirmed their anisotropic structure, with large (≈100–300 μm diameter) axially aligned macropores (Figures 2A and S2), similar to previous reports.

It has previously been demonstrated that uniform, close-packed, GO monolayer films can be assembled onto positively charged surfaces by an LbL strategy.^38^ This method relies on entropy gain upon expulsion of anions as negatively charged GO adsorbs onto positively charged surfaces.^39^ In water, CNF foams are negatively charged,^40^ suggesting that GO assembly onto the macropore walls in CNF foams could be possible, if GO was made positively charged (GO+). Modifying the CNF foams sequentially with GO+ and GO by an LbL-strategy offer several benefits. First, the LbL strategy affords films with higher uniformity than what is achievable by most other methods.^38,39,41,42^ Second, the sequential deposition allows for increased loading capacity of GO onto the foam.

To produce GO+, we adapted a previous literature protocol^43^ to attach branched polyethylenimine (b-PEI) to GO. This (b-PEI) cationic polymer is characterized by primary, secondary and tertiary amines separated by aliphatic CH_2_–CH_2_ spacers. The amine-groups in b-PEI make it straightforward to attach b-PEI to GO via nucleophilic addition to GO epoxy groups (Figure 2B).^44^ At neutral pH, the resulting GO+ had a zeta potential of ζ = +35 ± 5 mV (compared to ζ = −35 mV ± 9 mV for GO). No other zeta potential peak was observed for the GO+ suspension (Figure S3B), strongly suggesting that all GO converted to GO+ by attachment of b-PEI to GO. Furthermore, the zeta potentials of both GO and GO+ are sufficient to suggest colloidal stability over long time periods,^45,46^ which was confirmed over several months of storage (Figure S4).

**Figure 3:**
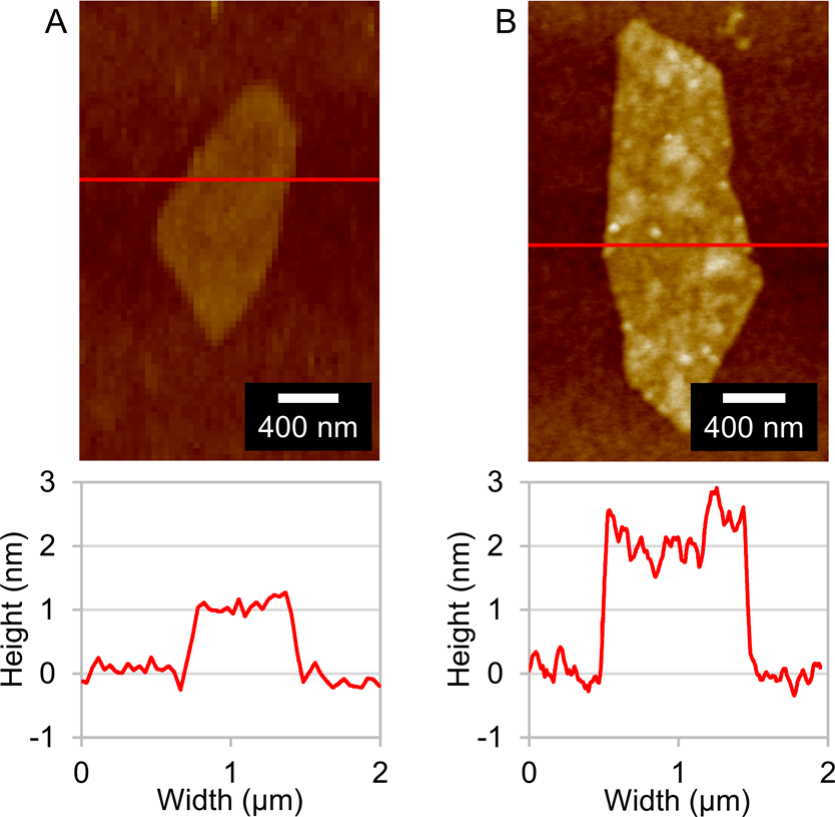
Typical AFM micrographs of (A) GO and (B) GO+ sheets deposited on Si wafers. Shown are also typical height profiles.

AFM characterization further confirmed successful attachment of b-PEI to GO, where single sheets of GO, deposited on Si wafers, showed an average height of 0.9 ± 0.1 nm (Figure 3A), while single sheets of GO+, showed an average height of 2.1 ± 0.2 nm (Figure 3B). Moreover, the average roughness of the GO sheets was  = 0.11 ± 0.02 nm, while for GO+, the average roughness was  = 0.36 ± 0.04 nm (Supporting Information, Section S2).

After production of anisotropic CNF foams and GO+, we turned to GO+ self-assembly into the foams. Our strategy is dependent on soaking the foams with aqueous suspensions of GO+, something that proved challenging. Upon adding water dropwise to the top of the foams (i.e., looking down the macropores), followed by drying at 95 °C, the foams contracted by collapse of the macropores (Figure S5A). This contraction is likely driven by surface tension as the water volume in the pores is gradually reduced during evaporation. In a first attempt to mitigate this problem, we affixed the foams to a Styrofoam plate using needles along the perimeter of the foam. However, upon evaporation of water from this fixed foam, the foam cracked instead of contracting (Figure S5B). After these failed attempts, we surmised that the surface tension of water (60 mN m^–1^ at 95 °C)^47^ is too high to allow for removal by drying.

Fortunately, both GO+ and GO are dispersible in methanol/water mixtures, at least in the short term. E.g., in 5:1 methanol/water, the GO+ and GO exhibited zeta potentials of ζ = +24 ± 8 mV and ζ = −25 ± 8 mV, respectively (Figure S3). In addition, our CNF is negatively charged in 5:1 methanol/water (ζ = −23 ± 2.2 mV, Figure S3), suggesting LbL assembly with GO+ may be possible. We therefore soaked the foams (by dropwise addition to the top of the foams, Figures 2C and S5C), first with pure methanol, and then with methanol/water mixtures of successively higher water volume fractions. Finally, GO+ in 5:1 methanol/water was added to the foams, followed by addition of GO in 5:1 methanol/water. The foams were then soaked with methanol/water mixtures of successively lower water content, until soaked in pure methanol. Methanol has a surface tension of only 20 mN m^–1^ at 55 °C,^48^ and no contraction or cracking of the foams was observed (Figures 2C, and S5C) after drying at 55 °C. After addition of GO+ and GO, the foams turned brown, indicating that GO+ and GO were retained in the foams (Figure 2C).

In the final step, the GO+/GO film was reduced to rGO using hydroiodic acid/acetic acid vapor, by a well-established protocol^49^ (Figure 2C). We also prepared CNF control foams from the same batch of native CNF foams that were used for rGO-CNF foams. The CNF control foams underwent identical impregnation, drying, and reduction protocols as the rGO-CNF foams (Figure S5D).

**Figure 4:**
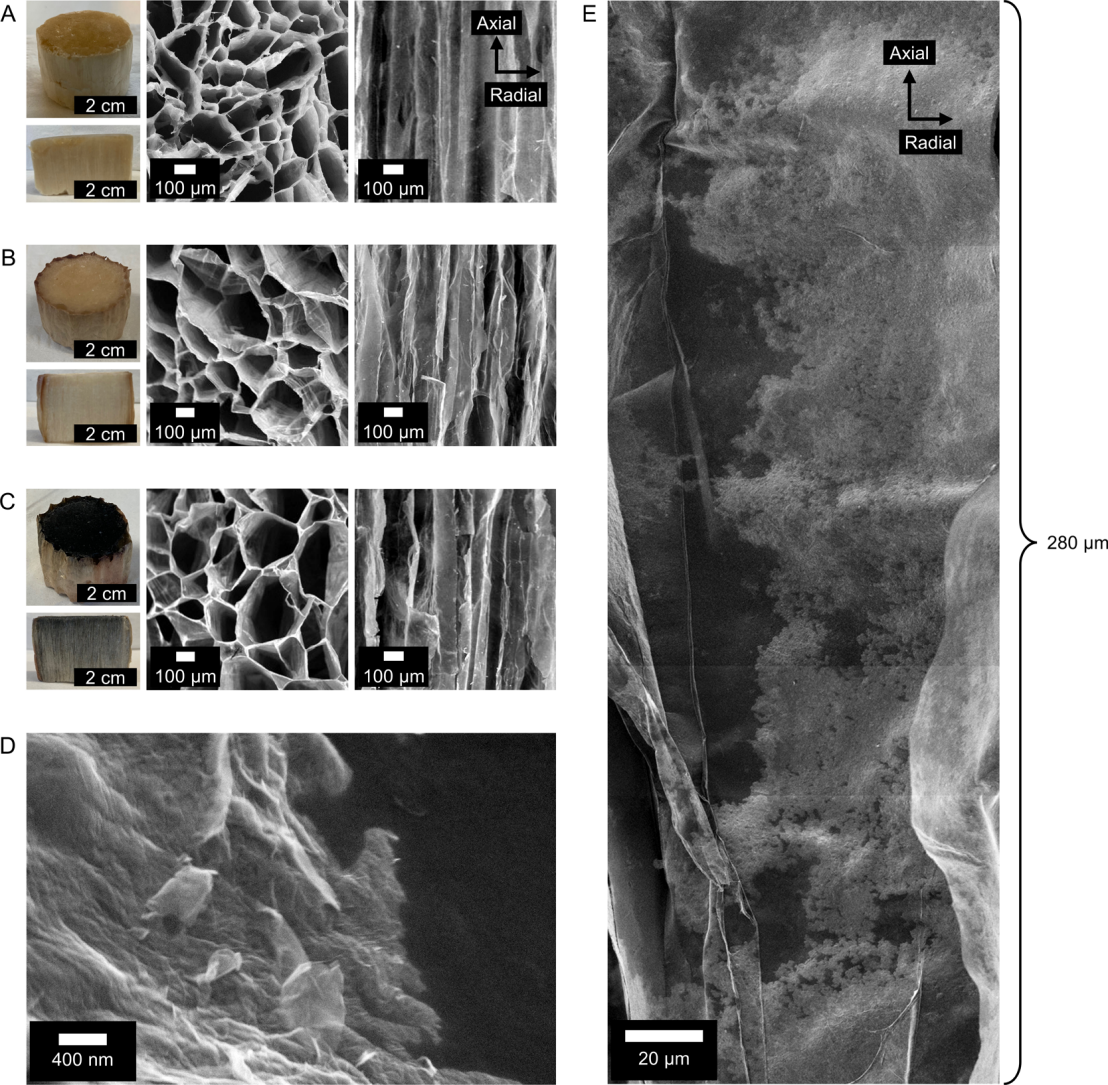
Structural characterization of rGO-CNF foams and CNF control foams. (A–C) Left to right: photo of foam and of foam cut along the axial direction, top view SEM micrograph, and side view SEM micrograph. (A) Native foam. (B) Anisotropic CNF control foam. (C) Anisotropic rGO-CNF foam. (D) High-resolution SEM micrograph of a macropore wall in an rGO-CNF foam. (E) FIB micrograph (stitched from five individual images), of a macropore wall in an rGO-CNF foam. In (D,E), bright areas correspond to rGO sheets, and darker areas correspond to the CNF-foam pore-wall. Please see Figure S6 for a FIB image of the CNF control foam without rGO.

CNF control foams and rGO-CNF foams were then thoroughly characterized by SEM (Figure 4A–D) and focused ion beam (FIB) microscopy (Figure 4E). SEM micrographs of the top of the foams clearly show that the cellular structure of the native anisotropic foams (Figure 4A) is retained in the CNF control, and rGO-CNF foams (Figure 4B,C). Importantly, SEM micrographs of axial cross sections also show that, after impregnation and drying from methanol, the anisotropic nature of the native foams (Figure 4A) is clearly retained, with elongated macropores extending several millimeters through the foams (Figure 4B,C).

To visualize individual rGO sheets in the foams, we turned to high-resolution SEM (Figure 4D) and FIB microscopy (Figure 4E) of cross sections, cut axially from the rGO-CNF foams. In Figure 4E, a micrograph of a typical macropore extending 280 μm in the axial direction has been generated by stitching several individual micrographs together. Two important observations can be made from this micrograph: first, there is a film adsorbed to the macropore wall (bright areas) that consists of individual sheet-like structures. Second, the film is mostly percolated, but discontinuities appear to exist. In high-resolution SEM micrographs (Figure 4D) individual rGO sheets are clearly observed, confirming that the bright areas in Figure 4E are indeed rGO.

FIB imaging of CNF control foams (Figure S6), showed no bright regions as observed in the rGO-CNF foams. In addition, during FIB imaging of CNF control foams, there was significant charging (Figure S6), also supporting^50^ the notion that an electrically conductive film had been introduced in the rGO-CNF foams, where no charging was observed (Figure 4E). However, both rGO-CNF and CNF control foams displayed low overall axial electrical conductivities (Table S5), corroborating the SEM and FIB observations that the rGO film in the rGO-CNF foams was not fully percolated.

### Thermal Conductivity of rGO-CNF and CNF Control Foams

Measurements to determine axial and radial thermal conductivities were carried out using the transient plane source (TPS) method^51,52^ in a Thermal Constants Analyzer TPS 2500 S (Hot Disk). Although frequently not accounted for, the relative humidity (RH) is known to strongly impact thermal conductivity of CNF foams,^9^ and we therefore adapted our Hot Disk instrument to allow control over RH during measurements.

**Figure 5:**
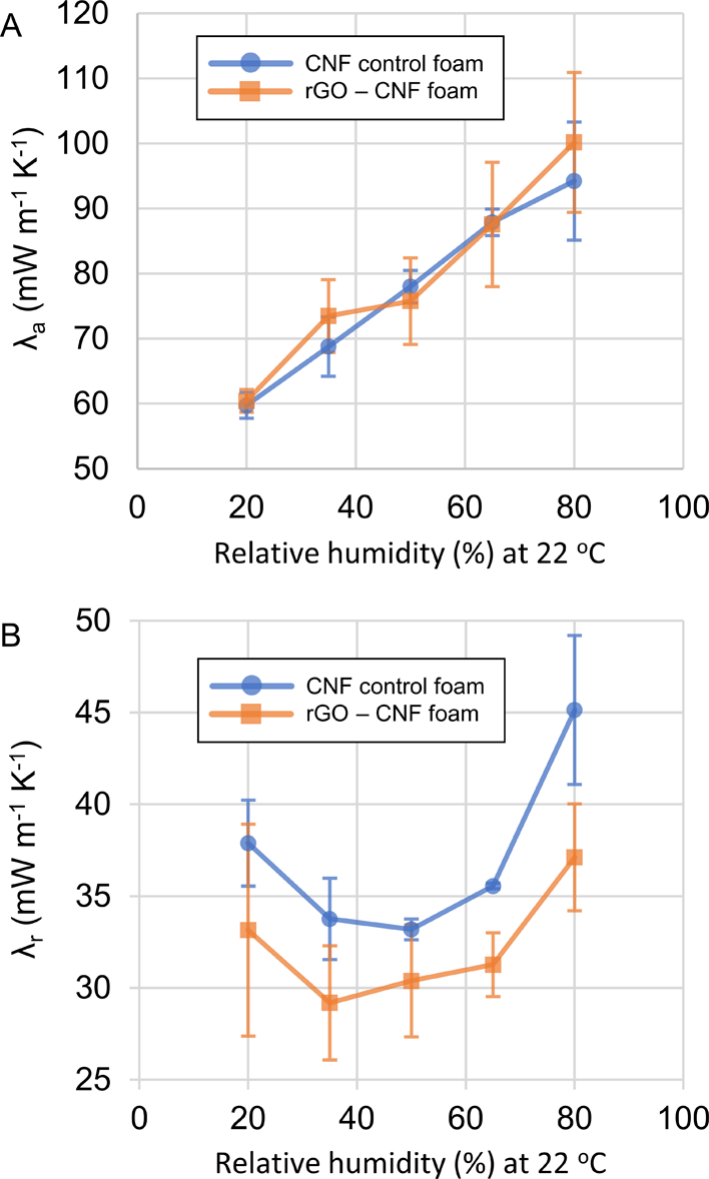
Axial and radial thermal conductivities of rGO-CNF (orange squares) and CNF control foams (blue circles) at 22 °C and different RH (%). Error bars are two standard deviations wide and based on measurement of three individual foam pairs. (A) Axial thermal conductivities (λ_a_). (B) Radial thermal conductivities (λ_r_).

As expected,^9,10^ the thermal conductivities in the foams were anisotropic, with λ_a_/λ_r_ ranging from 1.6 to 2.8. As previously observed^9,18,53^ in anisotropic CNF foams, λ_a_ increased monotonically with RH, and ranged from about 60 mW m^–1^ K^–1^ at 20% RH to about 100 mW m^–1^ K^–1^ at 80% RH for both the rGO – CNF and CNF control foams (Figure 5A). This monotonic increase in λ_a_ with RH is presumably due to increased solid conduction along the CNF due to increased amount of adsorbed water at higher RH.^9^ Also note that, within experimental error, we did not observe any significant difference in λ_a_ between the rGO-CNF and CNF control foams at any relative humidity (Figure 5A).

The radial thermal conductivity of the rGO-CNF and the CNF control foams ranged between 29 and 45 mW m^–1^ K^–1^, which is comparable to many insulating materials previously reported (see Table S4 for a comparison of our foams with previously reported materials). Importantly, while we see no significant difference in λ_a_ between the rGO-CNF foams and the CNF control foams, we do observe a difference in λ_r_. In fact, λ_r_ is lower in the rGO-CNF foams than in the CNF control foams at all relative humidity levels (Figure 5B). Taken together, the differences in λ_r_ at different RH suggest, with 99.5% significance (Supporting Section S3), that the rGO-CNF foams have lower radial thermal conductivity than the CNF control foams.

In the CNF control foams, λ_r_ displayed a dependence on RH with a minimum around 35–65% RH (Figure 5B), similar to previous studies.^9,10^ This U-shaped dependence of λ_r_ on RH has been thoroughly investigated in previous work.^9^ In this previous work, the dependence of λ_r_ on RH was attributed to competition between two opposing effects: At higher RH, fiber–fiber separation is increased, leading to increased phonon scattering which reduces λ_r_. However, at higher RH, there is also partial replacement of air with water in the mesopores, increasing λ_r_.^9^ Due to measurement uncertainty regarding λ_r_ in the rGO-CNF foams (Figure 5B), we cannot assertively state that the rGO-CNF foams show a similar (U-shaped) trend regarding the dependence of λ_r_ on RH. Thus, the rGO-CNF foams show lower λ_r_ than the CNF control foams at all relative humidities, but further study is needed to determine how rGO in CNF-foams influences the dependence of λ_r_ on relative humidity.

The rGO-CNF and CNF control foams were made from the same batch of native anisotropic CNF foams, and they were treated in an identical fashion through all processing steps, with the only difference that rGO was added to the rGO-CNF foams (please refer to the Experimental section). Therefore, it is not surprising that the rGO-CNF and CNF control foams show very similar physical characteristics: density, specific heat capacity, macropore size, number-density of macropores, BET surface area, overall porosity, and mesoporosity (Table S1, Figures S7–S12). Thus, the foams are essentially identical, save for the presence of uniaxially aligned rGO in the rGO-CNF foams. We therefore conclude that the lower λ_r_ in rGO-CNF foams is due to the uniaxially aligned rGO. Interestingly, GO has previously been introduced in anisotropic CNF foams, but during the freeze-casting step,^10^ likely resulting in a more random distribution of GO in the macropore walls. In this previous work, the introduction of GO (a good thermal conductor^33,54^) resulted in increased λ_r_,^10^ which is not surprising considering that sheets extending radially, through the macropore walls, would increase thermal conduction in this direction. Importantly, this previous work suggests that it is indeed the hierarchical ordering of rGO—aligned along the macropore walls—in our work that results in lower λ_r_.

We identify three possible mechanisms that may rationalize why λ_r_ is reduced in the rGO-CNF foams compared to the CNF control foams. In highly insulating materials, radiative thermal conduction is often non-negligible.^55^ Therefore, one plausible mechanism (i) involves suppression of the radial component of radiative thermal conduction. CNF is a poor absorber, but good emitter of thermal (infrared) radiation.^56,57^ In stark contrast, the infrared absorption is strong in rGO, and absorbed photons convert to phonons,^58,59^ which would then transport thermal energy in the axial direction.^33^ Thus, it is plausible that rGO suppresses the radial component of radiative thermal transport, thereby lowering λ_r_.

**Figure 6:**
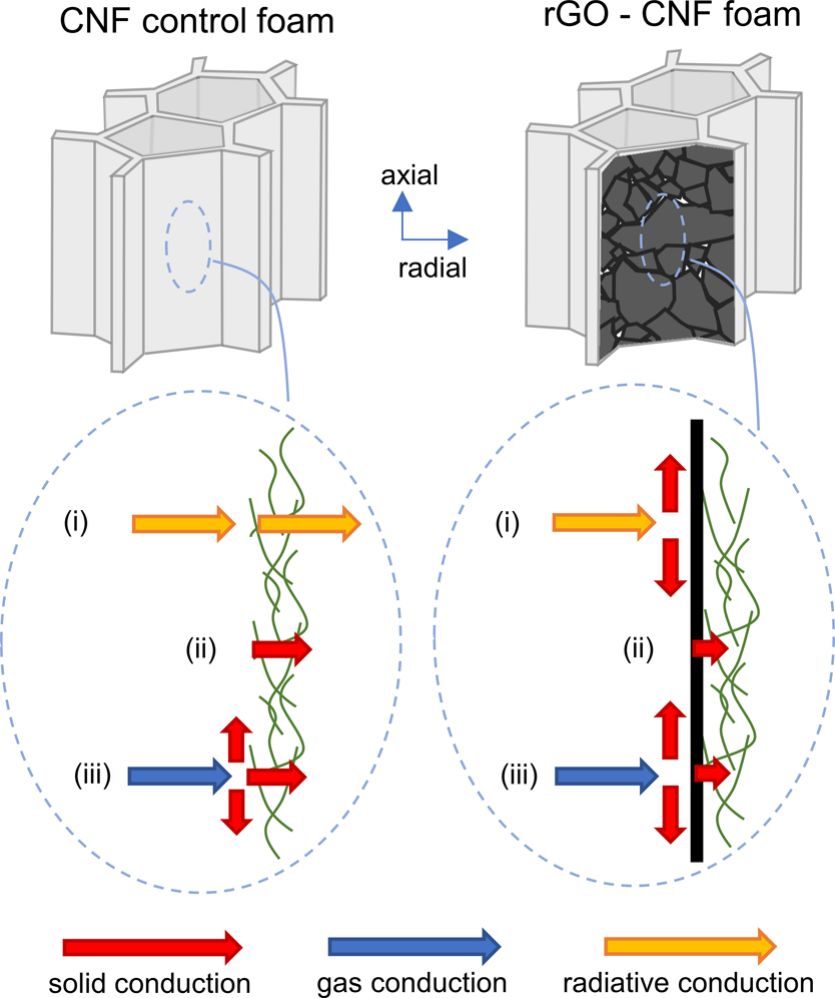
Schematic illustration of potential mechanisms for the observed lower λ_r_ in rGO-CNF foams compared to CNF control foams: (i) In rGO-CNF foams, the radial component of radiative thermal energy transfer may be efficiently converted into axially aligned phonons in rGO. (ii) The thermal boundary resistance at rGO/CNF interfaces may be larger than at CNF/CNF interfaces. (iii) In rGO-CNF foams, the radial component of gas kinetic energy may convert into axially aligned phonons more efficiently than in CNF control foams because rGO is aligned along the axial direction, and the in-plane phonon conduction in rGO is very high.

Phonon scattering at interfaces can dominate thermal conduction in nanoscale materials.^9,28,60−62^ Therefore, a second plausible mechanism (ii) is that the thermal boundary resistance at rGO/CNF interfaces is larger than at CNF/CNF interfaces, which would reduce λ_r_. Finally, because of the extremely high thermal conduction anisotropy in rGO,^33^ a film of aligned rGO sheets transports thermal energy, by phonons, very efficiently along the film-plane, but very poorly across the plane.^29−31,54^ Another plausible mechanism (iii) for reduction of λ_r_ in the rGO-CNF foam therefore involves conversion of the radial component of gas kinetic energy in the axial macropores to axially aligned phonons in rGO (Figure 6).

While mechanisms (i)–(iii) may be important to rationalize the observed reduction in λ_r_, further work is necessary to establish which of the mechanisms (if any) is dominating. However, note that if any of these mechanisms is important for the observed reduction in λ_r_, it suggests that obtaining a more percolated rGO–film, than what was achieved in this work (Figure 4E), could further reduce λ_r_. Also note that mechanism (i) and (iii) should be expected to lead to increased λ_a_ (in addition to lower λ_r_). As described above, we do not see such an increase in λ_a_ in the rGO-CNF foams compared to the CNF control foams (Figure 5A). Note, however, that if an increase in λ_a_ is on the same order as the decrease in λ_r_ (i.e., ≈5 mW m^–1^ K^–1^, Figure 5B), it could be obscured by the experimental standard deviations associated with the measurement of λ_a_ (which are larger than 5 mW m^–1^ K^–1^, Figure 5A).

Finally, while we have shown that λ_r_ is reduced upon introduction of rGO on the macropore walls of CNF foams, both our rGO-CNF and CNF control foams show higher radial thermal conductivities (≈29–45 mW m^–1^ K^–1^) than native anisotropic CNF foams (≈14–28 mW m^–1^ K^–1^).^9,10^ The exceptionally low thermal conductivity in native freeze cast CNF foams is thought to be, in large part, due to the anisotropic spatial arrangement of CNF, aligned in the axial direction.^9,10,20,22,24^ In addition, thermal conductivity depends strongly on foam density.^63,64^ We therefore speculate that the higher thermal conductivity in the rGO-CNF and CNF control foams compared to native anisotropic CNF foams may be due to densification and partial rearrangement of CNF in the macropore walls during cross-linking and wet impregnation. Thus, to fully make use of rGO to reduce λ_r_ in anisotropic CNF foams, it would be beneficial to develop methods to incorporate axially aligned rGO, without increasing the intrinsic radial conductivity of the native foam.

## Conclusions

We have shown that coating rGO onto the macropore walls of anisotropic CNF foams reduces their radial thermal conductivity. In our work, the rGO film is not completely percolated, but its introduction still yields a significant reduction in λ_r_. Developing protocols for better rGO-film percolation could therefore lead to further reduction in λ_r_. However, to make efficient use of rGO to reduce λ_r_, protocols for rGO deposition onto the macropore walls of CNF foams must be developed that do not realign or densify CNF in the foam walls.

LbL postmodification of anisotropic CNF foams has, to our knowledge, not been demonstrated before. We believe that our protocol could enable self-assembly postmodification with any species that is soluble/dispersible in methanol/water mixtures. Such species include (in addition to GO) conducting polymers,^65^ carbon nanotubes,^66^ catalytic nanoparticles^67^ etc., which thereby could enable postmodification of anisotropic CNF foams for applications in energy storage and conversion,^68^ gas sensing,^69^ heterogeneous catalysis,^70^ etc.

## Experimental Section

### Materials

Never-dried sulfite softwood pulp (Domsjö Fabriker AB) was used as the cellulose starting material. Sodium hypochlorite (12% in water) and TEMPO (2,2,6,6-tetramethyl-1-piperidinyloxy free radical, 98% purity) were obtained from Alfa Aesar. Sodium hydroxide (99.2% purity), hydrochloric acid (35%), and methanol (anhydrous) were obtained from VWR Chemicals. Sodium bromide (99.5% purity), BTCA (butane tetracarboxylic acid, 99% purity), SHP (sodium hypophosphite monohydrate, 99% purity), b-PEI (branched polyethylenimine, *M*_w_ = 750 kDa, 50 wt % in water), acetic acid (glacial, 99.7% purity), and hydroiodic acid (57%) were obtained from Sigma-Aldrich. GO (graphene oxide, 4 g L^–1^ in water) was obtained from Graphenea. Pure water (Milli-Q) was used for all experiments, unless stated otherwise.

### CNF Synthesis

The CNF synthesis was adopted from previous protocols.^15,35,36^ First, the cellulose pulp was washed using deionized (DI) water adjusted to pH = 2. This was done by stirring (500 rpm, 30 min), followed by vacuum filtration. The washing was repeated until the conductivity of the cellulose suspension was <5 μS. Then, 40 g (dry-basis) rinsed pulp was mixed with TEMPO (0.64 g), sodium bromide (4 g), and 1.87 L of DI water. The pH was set to 10 and maintained at this level throughout the oxidation process by adding aqueous NaOH (0.5 mol L^–1^). Sodium hypochlorite (60 mmol, dissolved in a small volume of DI water) was then added into the pulp mixture, which was left to stir (500 rpm) for 1 h. The oxidized pulp was then rinsed as described above until the conductivity was below 5 μS. Finally, the oxidized pulp was broken down using a high-pressure (1600 bar) microfluidizer (M-110EH, Microfluidics), with a series of chambers sized 200 and 100 μm. The suspension was cycled through the chambers eight times to produce TEMPO oxidized CNF.

### Production of Anisotropic CNF Foams

We adapted previous protocols^10,23^ for production of anisotropic CNF foams, and adapted our cross-linking protocol from a previous protocol describing cross-linking of isotropic CNF foams.^37^ An aqueous suspension of CNF (5 mg mL^–1^) was mixed with a high-speed disperser (Ultra-Turrax, IKA) at 10,000 rpm for 2 min. A cross-linker (BTCA) was then added at 9 wt % with respect to the CNF, followed by addition of SHP at 4 wt %. The suspension was again mixed at 10,000 rpm for 8 min, while the pH was set to 7, using aqueous NaOH (5 mol L^–1^). This suspension was then degassed on a Schlenk line and cooled to 0 °C on an ice bath. For directional freeze-casting, the suspension was poured into a Teflon-mold attached to a copper plate, and placed on a coldfinger kept at −135 °C (Figure 2A), and allowed to freeze for 60 min, before recovering the frozen suspension. To ensure that the axial macropores were open at both ends of the foams, the frozen suspensions were filed down while in the frozen state. From the bottom (where the suspension was in contact with the copper plate), 16 mm were removed by filing, while from the top, 2 mm was removed. The frozen suspension was then allowed to freeze-dry (Alpha 1–2 LD Plus, Martin Christ) at 0.045 mbar for 5 days. After freeze-drying, the foams were cross-linked in an oven at 170 °C for 10 min.

### GO+ Synthesis

GO+ was synthesized by modification of a previous protocol^43^ for amine functionalization. GO was first purified by repeated centrifugation to remove both small sheets and large/unexfoliated sheets. The GO suspension was centrifuged at 22,800 rcf (Fiberlite F15-8 × 50cy rotor, Sorvall Lynx 6000, Thermo Fisher Scientific) for 20 min, followed by removal of the supernatant and redispersion of the sediment in water (this step was repeated three times). Finally, the suspension was centrifuged at 1050 rcf (10 min) and the supernatant was collected. The resulting GO stock dispersion (0.5 g L^–1^) was then used to produce GO+. First, 100 mL water and 0.5 mL b-PEI (*M*_w_ = 750 kDa, 50 wt % in water) was mixed in a reaction flask. The pH was then adjusted to 11.5 using 8 mL aqueous NaOH (0.5 mol L^–1^). Then, 50 mL of the GO stock was added, and the dispersion stirred for 40 min. To precipitate the GO+ product, 35 mL aqueous HCl (0.5 mol L^–1^) was added (changing the pH to 1), and the dispersion stirred for another 5 min. The precipitated GO+ product was then purified by repeated centrifugation at 22,800 rcf (5 min) with redispersion of the sediment in pure water. This purification was repeated 3 times before redispersion in pure water with a concentration (based on GO) of 0.5 mg mL^–1^. The pH in the resulting GO+ stock dispersion was 7. This dispersion had a zeta potential of ζ = +35 ± 5 mV, indicating both that b-PEI was successfully attached to GO, and that the resulting GO+ is colloidally stable.^45,46^

### GO+ and GO Self-Assembly (LbL) in Anisotropic CNF Foams

First, GO+ and GO dispersions in 5:1 methanol/water were prepared; 2.5 mL of aqueous GO+ (or GO) stock dispersion was diluted with 12.5 mL methanol and thoroughly mixed using a volumetric pipet. The dispersion was then sonicated for 3 h. The concentration of GO+ (or GO) in these dispersions was 0.08 mg mL^–1^ (based on GO). For impregnation, a native anisotropic CNF foam was affixed to a Styrofoam plate using needles along the perimeter of the foam. Paper tissue was also placed between the foam and Styrofoam plate (Figure S5C). The foam was then soaked by dropwise addition to the top of the foam (Figures 2C and S5C), first with pure methanol, and then with methanol/water mixtures of successively higher water fractions. To avoid large surface tension gradients between additions, the methanol/water ratios were chosen so that the change in surface tension occurred linearly as the water fraction changed (Table S2). We did this because we surmised that large surface tension gradients in the foams during impregnation could deform the foams. After fully soaking the foam in 5:1 methanol/water, GO+ in 5:1 methanol/water was added, followed by addition of GO (in 5:1 methanol/water). The foams were then soaked with methanol/water mixtures of successively lower water content, until the foams were completely soaked in pure methanol. Finally, the foams were placed in an oven at 55 °C to dry for 20 h. Anisotropic CNF control foams were impregnated with methanol/water mixtures in the same fashion as described above, but without addition of GO+ or GO (Figure S5D and Table S3). The same batch of native CNF foams were used for both rGO-CNF and CNF control foams.

### Reduction of GO+/GO-CNF Foams to rGO-CNF Foams

We adapted a literature protocol^49^ for vapor reduction of the GO+/GO thin films inside the anisotropic CNF foams. First, 60 mL of acetic acid (glacial) was added to a glass desiccator. Then, 24 mL of hydroiodic acid (57%) was added, and thoroughly mixed with the acetic acid. The foams were then quickly added onto a stage in the desiccator, making sure they were not in contact with the acetic acid/hydroiodic acid mixture. The desiccator was then sealed with vacuum grease and put in an oven at 40 °C for 23 h. Finally, the foams were taken from the desiccator and placed in the oven at 55 °C for another 23 h, to remove residual acetic acid/hydroiodic acid vapor. After this treatment, the GO+/GO-CNF foams turned black (thus forming rGO-CNF foams). CNF control foams were treated in the same fashion as the rGO-CNF foams (all foams were placed together in the same desiccator).

### Estimation of the Specific Heat Capacity (*C*_P,wet_) at Different RH

Determination of the specific heat capacity of the dry foams (*C*_P,dry_) was done by differential scanning calorimetry (DSC), using a Mettler Toledo 820 calorimeter. Measurements were done under N_2_ at 0% RH, and a temperature range of [−20, 50] °C with a heating rate of 10 K min^–1^. Three independent measurements were used for both rGO-CNF and CNF control foams. For each measurement, 10 mg of dry foam (dried at 105 °C for 24 h) was placed in an aluminum crucible with a pierced lid. The crucible was again dried for 24 h at 105 °C before DSC measurement. An empty crucible was used as reference and sapphire was used as a standard. Estimations of *C*_P,wet_ at each RH were then done by measuring the moisture uptake *w*_H_2_O_ into the foams at different RH, then using the rule of mixtures (eq 1) to estimate *C*_P,wet_ at each RH.^9^
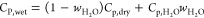
1

The moisture uptake into the foams was measured under controlled RH at 295 K. The foams were placed on a precision balance (BP 210 S, Sartorius) inside a humidity chamber (CLIMACELL 111-EVO, MMM Medcenter), and the weight of the foams was recorded as a function of RH (at 20, 35, 50, 65, 80%), thereby determining the RH dependent weight fraction of water (*w*_H_2_O_). The foam was allowed to equilibrate with the water vapor for 6 h at each RH, which ensured the water sorption reached a steady value. The dry weight of the foams was determined by weighing the foams while drying at 105 °C.

### Estimation of the Density (ρ_foam_) of the Foams at Different RH

The density of the dry foams was determined measuring the dry weight of the foams (see above) and by measuring the volume of the foams (*h*π*r*^2^), where *h* is the height of the foam, and *r* is the radius. The RH dependent foam densities were then determined by measuring the volume of the foams and the weight of the foams at each RH (see above). No RH dependent volume change in the foams was observed during these measurements. RH dependent densities for both the rGO-CNF foams and the CNF control foams are presented in Table S6.

### Estimation of % Overall Porosity (Π)

The % porosity of the dry foams was calculated as . Here, ρ_CNE_ = 1460 kg m^–3^ was used.^10^ The contribution of rGO to the density in rGO-CNF foams was neglected, because rGO is only present at about 2 wt % in the foams. The porosity of the rGO-CNF and CNF control foams is presented in Table S1.

### Thermal Conductivity Measurements of CNF Control Foams and rGO-CNF Foams

Thermal conductivities (λ_a_ and λ_r_) were determined with a TPS 2500 S Hot Disk Thermal Constant Analyzer (Hot-disk AB) in the anisotropic mode. A Kapton transient plane source sensor (6.4 mm in diameter) was placed between two identical foams (diameter: 2.8 ± 0.1 cm, height: 2 ± 0.2 cm), and a small weight (61 g) was placed on the foams to ensure good contact between sensor and foams. The foams used for thermal conductivity measurements have a cylindrical shape (as seen in Figure 2A,C). The top of the foams (i.e., the side to which GO+ and GO solutions were added during LbL assembly, see above) was always placed facing the sensor, to ensure reproducibility of the results. This setup was placed inside a custom-built chamber, allowing control over RH. Five measurements were done at 15 min intervals for each RH at 295 K. A heating power of 10 mW and a measurement time of 10 s was used. Both rGO-CNF and CNF control foams were investigated using 3 individual foam pairs. Determinations of λ_a_ and λ_r_ were done using the Hot Disk software under consideration of the wet specific heat capacity (*C*_P,wet_) and density of the foams at each RH, determined as described above.

### AFM

The height and length of our CNF as well as the roughness and height of our GO and GO+ were determined using a Multimode-8 AFM (Bruker) operating in peak-force tapping mode with the ScanAsyst automatic optimization algorithm. A drop of dilute aqueous CNF dispersion was dried on freshly cleaved mica before imaging. For the GO/GO+ characterization, a drop of dilute GO or GO+ in 5:1 methanol/water was deposited on a Si wafer. Data treatment was conducted in Nanoscope Analysis 2.0 and ImageJ.

### Zeta Potential

Zeta potential measurements were carried out using a Zetasizer Nano ZS90 (Malvern Instruments), equipped with a dip cell.

### SEM

Micrographs in Figures 2, 4A–C, S2, and S7–S10, were collected on a TM 3000 tabletop scanning electron microscope (Hitachi High Technologies). The microscope is equipped with a backscattered electron detector and was operated at an accelerating voltage of 15 kV. Micrographs in Figures 4D,E and S6 were collected on a Zeiss Crossbeam 550 FIB-SEM instrument. Images were collected at an extra high tension (EHT) of 5 kV and a focused ion beam (FIB) of 30 kV.

### Conductometric Titration

Conductometric titration was carried out using sodium hydroxide as the titrant, determining the surface charge of our CNF to 0.56 mmol COO^–^ per gram CNF.

### BET

To determine the surface areas of our rGO-CNF foams and CNF control foams, Brunauer–Emmett–Teller (BET) analysis was employed on N_2_ adsorption/desorption isotherms. Pore distributions were assessed using the BJH model on the N_2_ adsorption/desorption isotherms. About 0.5 g was used for each determination (corresponding to three individual foams that were cut up and squeezed together to remove the macroporosity). The measurements were carried out at a temperature of −196 °C using a Micrometrics ASAP 2020 instrument (Micromeritics Instrument Corporation). Before the analysis, the sample was subjected to degassing at 105 °C for a duration of 12 h.

### Electrical Conductivity

The electrical conductivity of the foams was measured using a two-point measurement method with copper (plate) electrodes and a Keithley 2400 SourceMeter. The foams were used as is, while the electrode surfaces were cleaned and polished to reduce contact resistance. A constant load and voltage were applied to the sample, and the current was recorded multiple times to ensure data reliability. The resistance was calculated using Ohm’s law, and the electrical conductivity was determined as the reciprocal of the resistivity, which was calculated based on the sample’s geometry.

## Supporting Information Available

Additional figures showing image statistics, zeta potential measurements, FIB micrograph of CNF control foam, N_2_ adsorption/desorption isotherms; additional tables presenting physical properties of the foams, additional experimental details, compilation of thermal conductivity data for different materials reported in previous literature, electrical conductivities of rGO-CNF and CNF control foams, relative humidity dependent densities of rGO-CNF and CNF control foams; statistical treatment of thermal conductivity data (PDF)

## References

[ref1] International Energy Agency Transition to Sustainable Buildings: Strategies and Opportunities to 2050; OECD/IEA, 2013.

[ref2] International Energy Agency and the United Nations Environment Programme. 2018 Global Status Report: Towards a Zero-Emission, Efficient and Resilient Buildings and Construction Sector; International Energy Agency, 2018.

[ref3] Tracking Clean Energy Progress 2017; International Energy Agency, 2017.

[ref4] Jiang X.; Zhao Z.; Zhou S.; Zou H.; Liu P. Anisotropic and Lightweight Carbon/Graphene Composite Aerogels for Efficient Thermal Insulation and Electromagnetic Interference Shielding. ACS Appl. Mater. Interfaces 2022, 14 (40), 45844–45852. 10.1021/acsami.2c13000. 36166730

[ref5] Tan Z.; Yoo C. G.; Yang D.; Liu W.; Qiu X.; Zheng D. Rigid-Flexible” Anisotropic Biomass-Derived Aerogels with Superior Mechanical Properties for Oil Recovery and Thermal Insulation. ACS Appl. Mater. Interfaces 2023, 15 (35), 42080–42093. 10.1021/acsami.3c07713. 37624365

[ref6] Xiong Z.; Marconnet A.; Ruan X. Unconventional and Dynamically Anisotropic Thermal Conductivity in Compressed Flexible Graphene Foams. ACS Appl. Mater. Interfaces 2022, 14 (43), 48960–48966. 10.1021/acsami.2c10880. 36256868

[ref7] Garemark J.; Perea-Buceta J. E.; Rico Del Cerro D.; Hall S.; Berke B.; Kilpeläinen I.; Berglund L. A.; Li Y. Nanostructurally Controllable Strong Wood Aerogel toward Efficient Thermal Insulation. ACS Appl. Mater. Interfaces 2022, 14 (21), 24697–24707. 10.1021/acsami.2c04584. 35511115 PMC9164199

[ref8] Berglund L.; Nissilä T.; Sivaraman D.; Komulainen S.; Telkki V. V.; Oksman K. Seaweed-Derived Alginate-Cellulose Nanofiber Aerogel for Insulation Applications. ACS Appl. Mater. Interfaces 2021, 13 (29), 34899–34909. 10.1021/acsami.1c07954. 34255967 PMC8323098

[ref9] Apostolopoulou-Kalkavoura V.; Hu S.; Lavoine N.; Garg M.; Linares M.; Munier P.; Zozoulenko I.; Shiomi J.; Bergström L. Humidity-Dependent Thermal Boundary Conductance Controls Heat Transport of Super-Insulating Nanofibrillar Foams. Matter 2021, 4 (1), 276–289. 10.1016/j.matt.2020.11.007.

[ref10] Wicklein B.; Kocjan A.; Salazar-Alvarez G.; Carosio F.; Camino G.; Antonietti M.; Bergström L. Thermally Insulating and Fire-Retardant Lightweight Anisotropic Foams Based on Nanocellulose and Graphene Oxide. Nat. Nanotechnol. 2015, 10, 277–283. 10.1038/nnano.2014.248. 25362476

[ref11] Jelle B. P. Traditional, State-of-the-Art and Future Thermal Building Insulation Materials and Solutions - Properties, Requirements and Possibilities. Energy Build. 2011, 43, 2549–2563. 10.1016/j.enbuild.2011.05.015.

[ref12] Zhou S.; Apostolopoulou-Kalkavoura V.; Tavares da Costa M. V.; Bergström L.; Strømme M.; Xu C. Elastic Aerogels of Cellulose Nanofibers@Metal-Organic Frameworks for Thermal Insulation and Fire Retardancy. Nano-Micro Lett. 2020, 12 (1), 9 10.1007/s40820-019-0343-4. PMC7770683 34138073

[ref13] Ding H.; Qiu S.; Wang X.; Song L.; Hu Y. Highly Flame Retardant, Low Thermally Conducting, and Hydrophobic Phytic Acid-Guanazole-Cellulose Nanofiber Composite Foams. Cellulose 2021, 28 (15), 9769–9783. 10.1007/s10570-021-04159-0.

[ref14] Guo L.; Chen Z.; Lyu S.; Fu F.; Wang S. Highly Flexible Cross-Linked Cellulose Nanofibril Sponge-like Aerogels with Improved Mechanical Property and Enhanced Flame Retardancy. Carbohydr. Polym. 2018, 179, 333–340. 10.1016/j.carbpol.2017.09.084. 29111059

[ref15] Gordeyeva K. S.; Fall A. B.; Hall S.; Wicklein B.; Bergström L. Stabilizing Nanocellulose-Nonionic Surfactant Composite Foams by Delayed Ca-Induced Gelation. J. Colloid Interface Sci. 2016, 472, 44–51. 10.1016/j.jcis.2016.03.031. 27003498

[ref16] Li J.; Lu Z.; Xie F.; Huang J.; Ning D.; Zhang M. Highly Compressible, Heat-Insulating and Self-Extinguishing Cellulose Nanofiber/Aramid Nanofiber Nanocomposite Foams. Carbohydr. Polym. 2021, 261, 117837 10.1016/j.carbpol.2021.117837. 33766337

[ref17] Nguyen S. T.; Feng J.; Ng S. K.; Wong J. P. W.; Tan V. B. C.; Duong H. M. Advanced Thermal Insulation and Absorption Properties of Recycled Cellulose Aerogels. Colloids Surf., A 2014, 445, 128–134. 10.1016/j.colsurfa.2014.01.015.

[ref18] Kriechbaum K.; Apostolopoulou-Kalkavoura V.; Munier P.; Bergström L. Sclerotization-Inspired Aminoquinone Cross-Linking of Thermally Insulating and Moisture-Resilient Biobased Foams. ACS Sustain. Chem. Eng. 2020, 8 (47), 17408–17416. 10.1021/acssuschemeng.0c05601. 33344097 PMC7737238

[ref19] Zhao S.; Zhang Z.; Sèbe G.; Wu R.; Rivera Virtudazo R. V.; Tingaut P.; Koebel M. M. Multiscale Assembly of Superinsulating Silica Aerogels within Silylated Nanocellulosic Scaffolds: Improved Mechanical Properties Promoted by Nanoscale Chemical Compatibilization. Adv. Funct. Mater. 2015, 25 (15), 2326–2334. 10.1002/adfm.201404368.

[ref20] Chaudary A.; Patoary M. K.; Zhang M.; Chudhary T.; Farooq A.; Liu L. Structurally Integrated Thermal Management of Isotropic and Directionally Ice-Templated Nanocellulose/Chitosan Aerogels. Cellulose 2022, 29 (15), 8265–8282. 10.1007/s10570-022-04781-6.

[ref21] Munier P.; Apostolopoulou-Kalkavoura V.; Persson M.; Bergström L. Strong Silica-Nanocellulose Anisotropic Composite Foams Combine Low Thermal Conductivity and Low Moisture Uptake. Cellulose 2020, 27 (18), 10825–10836. 10.1007/s10570-019-02912-0.

[ref22] Kriechbaum K.; Munier P.; Apostolopoulou-Kalkavoura V.; Lavoine N. Analysis of the Porous Architecture and Properties of Anisotropic Nanocellulose Foams: A Novel Approach to Assess the Quality of Cellulose Nanofibrils (CNFs). ACS Sustain. Chem. Eng. 2018, 6 (9), 11959–11967. 10.1021/acssuschemeng.8b02278.

[ref23] Munier P.; Gordeyeva K.; Bergström L.; Fall A. B. Directional Freezing of Nanocellulose Dispersions Aligns the Rod-Like Particles and Produces Low-Density and Robust Particle Networks. Biomacromolecules 2016, 17 (5), 1875–1881. 10.1021/acs.biomac.6b00304. 27071304

[ref24] Antlauf M.; Boulanger N.; Berglund L.; Oksman K.; Andersson O. Thermal Conductivity of Cellulose Fibers in Different Size Scales and Densities. Biomacromolecules 2021, 22 (9), 3800–3809. 10.1021/acs.biomac.1c00643. 34510907 PMC8441976

[ref25] Dri F. L.; Shang S. L.; Hector L. G.; Saxe P.; Liu Z. K.; Moon R. J.; Zavattieri P. D. Anisotropy and temperature dependence of structural, thermodynamic, and elastic properties of crystalline cellulose I_β_: a first-principles investigation. Modell. Simul. Mater. Sci. Eng. 2014, 22 (8), 085012 10.1088/0965-0393/22/8/085012.

[ref26] Diaz J. A.; Ye Z.; Wu X.; Moore A. L.; Moon R. J.; Martini A.; Boday D. J.; Youngblood J. P. Thermal Conductivity in Nanostructured Films: From Single Cellulose Nanocrystals to Bulk Films. Biomacromolecules 2014, 15 (11), 4096–4101. 10.1021/bm501131a. 25286405

[ref27] Isogai A.; Saito T.; Fukuzumi H. TEMPO-Oxidized Cellulose Nanofibers. Nanoscale 2011, 3, 71–85. 10.1039/C0NR00583E. 20957280

[ref28] Apostolopoulou-Kalkavoura V.; Munier P.; Dlugozima L.; Heuthe V. L.; Bergström L. Effect of Density, Phonon Scattering and Nanoporosity on the Thermal Conductivity of Anisotropic Cellulose Nanocrystal Foams. Sci. Rep. 2021, 11, 18685 10.1038/s41598-021-98048-y. 34548539 PMC8455657

[ref29] Han N.; Viet Cuong T.; Han M.; Deul Ryu B.; Chandramohan S.; Bae Park J.; Hye Kang J.; Park Y. J.; Bok Ko K.; Yun Kim H.; Kyu Kim H.; Hyoung Ryu J.; Katharria Y. S.; Choi C. J.; Hong C. H. Improved Heat Dissipation in Gallium Nitride Light-Emitting Diodes with Embedded Graphene Oxide Pattern. Nat. Commun. 2013, 4, 1452 10.1038/ncomms2448. 23385596

[ref30] Yan Z.; Liu G.; Khan J. M.; Balandin A. A. Graphene Quilts for Thermal Management of High-Power GaN Transistors. Nat. Commun. 2012, 3, 827 10.1038/ncomms1828. 22569371

[ref31] Subrina S.; Kotchetkov D.; Balandin A. A. Heat Removal in Silicon-on-Insulator Integrated Circuits with Graphene Lateral Heat Spreaders. IEEE Electron Device Lett. 2009, 30 (12), 1281–1283. 10.1109/LED.2009.2034116.

[ref32] Li T.; Song J.; Zhao X.; Yang Z.; Pastel G.; Xu S.; Jia C.; Dai J.; Chen C.; Gong A.; Jiang F.; Yao Y.; Fan T.; Yang B.; Wågberg L.; Yang R.; Hu L. Anisotropic, Lightweight, Strong, and Super Thermally Insulating Nanowood with Naturally Aligned Nanocellulose. Sci. Adv. 2018, 4, eaar3724 10.1126/sciadv.aar3724. 29536048 PMC5844708

[ref33] Renteria J. D.; Ramirez S.; Malekpour H.; Alonso B.; Centeno A.; Zurutuza A.; Cocemasov A. I.; Nika D. L.; Balandin A. A. Strongly Anisotropic Thermal Conductivity of Free-Standing Reduced Graphene Oxide Films Annealed at High Temperature. Adv. Funct. Mater. 2015, 25 (29), 4664–4672. 10.1002/adfm.201501429.

[ref34] Yang W.; Zhao Z.; Wu K.; Huang R.; Liu T.; Jiang H.; Chen F.; Fu Q. Ultrathin Flexible Reduced Graphene Oxide/Cellulose Nanofiber Composite Films with Strongly Anisotropic Thermal Conductivity and Efficient Electromagnetic Interference Shielding. J. Mater. Chem. C 2017, 5 (15), 3748–3756. 10.1039/C7TC00400A.

[ref35] Saito T.; Kimura S.; Nishiyama Y.; Isogai A. Cellulose Nanofibers Prepared by TEMPO-Mediated Oxidation of Native Cellulose. Biomacromolecules 2007, 8 (8), 2485–2491. 10.1021/bm0703970. 17630692

[ref36] Saito T.; Isogai A. TEMPO-Mediated Oxidation of Native Cellulose. The Effect of Oxidation Conditions on Chemical and Crystal Structures of the Water-Insoluble Fractions. Biomacromolecules 2004, 5 (5), 1983–1989. 10.1021/bm0497769. 15360314

[ref37] Hamedi M.; Karabulut E.; Marais A.; Herland A.; Nyström G.; Wågberg L. Nanocellulose Aerogels Functionalized by Rapid Layer-by-Layer Assembly for High Charge Storage and Beyond. Angew. Chem., Int. Ed. 2013, 125 (46), 12260–12264. 10.1002/anie.201305137. 24573788

[ref38] Holm A.; Kunz L.; Riscoe A. R.; Kao K. C.; Cargnello M.; Frank C. W. General Self-Assembly Method for Deposition of Graphene Oxide into Uniform Close-Packed Monolayer Films. Langmuir 2019, 35 (13), 4460–4470. 10.1021/acs.langmuir.8b03994. 30836748

[ref39] Bertrand P.; Jonas A.; Laschewsky A.; Legras R. Ultrathin polymer coatings by complexation of polyelectrolytes at interfaces: suitable materials, structure and properties. Macromol. Rapid Commun. 2000, 21, 319–348. 10.1002/(SICI)1521-3927(20000401)21:7<319::AID-MARC319>3.0.CO;2-7.

[ref40] Beluns S.; Gaidukovs S.; Platnieks O.; Gaidukova G.; Mierina I.; Grase L.; Starkova O.; Brazdausks P.; Thakur V. K. From Wood and Hemp Biomass Wastes to Sustainable Nanocellulose Foams. Ind. Crops Prod. 2021, 170, 113780 10.1016/j.indcrop.2021.113780.

[ref41] Richardson J. J.; Cui J.; Björnmalm M.; Braunger J. A.; Ejima H.; Caruso F. Innovation in Layer-by-Layer Assembly. Chem. Rev. 2016, 116, 14828–14867. 10.1021/acs.chemrev.6b00627. 27960272

[ref42] Fuzzy Nanoassemblies—toward Layerd Polymeric Multicomposites.

[ref43] Bourlinos A. B.; Gournis D.; Petridis D.; Szabó T.; Szeri A.; Dékány I. Graphite Oxide: Chemical Reduction to Graphite and Surface Modification with Primary Aliphatic Amines and Amino Acids. Langmuir 2003, 19 (15), 6050–6055. 10.1021/la026525h.

[ref44] Yuan B.; Wang M.; Wang B.; Yang F.; Quan X.; Tang C. Y.; Dong Y. Cross-Linked Graphene Oxide Framework Membranes with Robust Nano-Channels for Enhanced Sieving Ability. Environ. Sci. Technol. 2020, 54 (23), 15442–15453. 10.1021/acs.est.0c05387. 33185431

[ref45] Everett D. H. Basic Principles of Colloid Science; The Royal Society of Chemistry: London, 1988.

[ref46] Li D.; Müller M. B.; Gilje S.; Kaner R. B.; Wallace G. G. Processable Aqueous Dispersions of Graphene Nanosheets. Nat. Nanotechnol. 2008, 3, 101–105. 10.1038/nnano.2007.451. 18654470

[ref47] Vargaftik N. B.; Volkov B. N.; Voljak L. D. International Tables of the Surface Tension of Water. J. Phys. Chem. Ref. Data 1983, 12, 817–820. 10.1063/1.555688.

[ref48] Jasper J. J. The Surface Tension of Pure Liquid Compounds. J. Phys. Chem. Ref. Data 1972, 1 (4), 841–1010. 10.1063/1.3253106.

[ref49] Moon I. K.; Lee J.; Ruoff R. S.; Lee H. Reduced Graphene Oxide by Chemical Graphitization. Nat. Commun. 2010, 1, 73 10.1038/ncomms1067. 20865806

[ref50] Kim K. H.; Akase Z.; Suzuki T.; Shindo D. Charging Effects on SEM/SIM Contrast of Metal/Insulator System in Various Metallic Coating Conditions. Mater. Trans. 2010, 51 (6), 1080–1083. 10.2320/matertrans.M2010034.

[ref51] Gustafsson S. E.; Karawackif E.; Khan N. Transient Hot-Strip Method for Simultaneously Measuring Thermal Conductivity and Thermal Diffusivity of Solids and Fluids. J. Phys. D: Appl. Phys. 1979, 12, 1411 10.1088/0022-3727/12/9/003.

[ref52] Gustafsson S. E.; Karawacki E.; Khan M. N. Determination of the Thermal-Conductivity Tensor and the Heat Capacity of Insulating Solids with the Transient Hot-Strip Method. J. Appl. Phys. 1981, 52 (4), 2596–2600. 10.1063/1.329068.

[ref53] Church T. L.; Kriechbaum K.; Schiele C.; Apostolopoulou-Kalkavoura V.; Hadi S. E.; Bergström L. A Stiff, Tough, and Thermally Insulating Air- and Ice-Templated Plant-Based Foam. Biomacromolecules 2022, 23 (6), 2595–2602. 10.1021/acs.biomac.2c00313. 35621041 PMC9198970

[ref54] Mu X.; Wu X.; Zhang T.; Go D. B.; Luo T. Thermal Transport in Graphene Oxide - From Ballistic Extreme to Amorphous Limit. Sci. Rep. 2014, 4, 3909 10.1038/srep03909. 24468660 PMC3904152

[ref55] Wang M.; Pan N. Modeling and Prediction of the Effective Thermal Conductivity of Random Open-Cell Porous Foams. Int. J. Heat Mass Transfer 2008, 51 (5–6), 1325–1331. 10.1016/j.ijheatmasstransfer.2007.11.031.

[ref56] Chen Y.; Dang B.; Fu J.; Wang C.; Li C.; Sun Q.; Li H. Cellulose-Based Hybrid Structural Material for Radiative Cooling. Nano Lett. 2021, 21 (1), 397–404. 10.1021/acs.nanolett.0c03738. 33301320

[ref57] Li T.; Zhai Y.; He S.; Gan W.; Wei Z.; Heidarinejad M.; Dalgo D.; Mi R.; Zhao X.; Song J.; Dai J.; Chen C.; Aili A.; Vellore A.; Martini A.; Yang R.; Srebric J.; Yin X.; Hu L. A Radiative Cooling Structural Material. Science 2019, 364 (6442), 760–763. 10.1126/science.aau9101. 31123132

[ref58] Bae J. J.; Yoon J. H.; Jeong S.; Moon B. H.; Han J. T.; Jeong H. J.; Lee G. W.; Hwang H. R.; Lee Y. H.; Jeong S. Y.; Lim S. C. Sensitive Photo-Thermal Response of Graphene Oxide for Mid-Infrared Detection. Nanoscale 2015, 7 (38), 15695–15700. 10.1039/C5NR04039F. 26350352

[ref59] Acik M.; Lee G.; Mattevi C.; Chhowalla M.; Cho K.; Chabal Y. J. Unusual Infrared-Absorption Mechanism in Thermally Reduced Graphene Oxide. Nat. Mater. 2010, 9 (10), 840–845. 10.1038/nmat2858. 20852618

[ref60] Chen G. Phonon Heat Conduction in Nanostructures. Int. J. Therm. Sci. 2000, 39 (4), 471–480. 10.1016/S1290-0729(00)00202-7.

[ref61] Pernot G.; Stoffel M.; Savic I.; Pezzoli F.; Chen P.; Savelli G.; Jacquot A.; Schumann J.; Denker U.; Mönch I.; Deneke C.; Schmidt O. G.; Rampnoux J. M.; Wang S.; Plissonnier M.; Rastelli A.; Dilhaire S.; Mingo N. Precise Control of Thermal Conductivity at the Nanoscale through Individual Phonon-Scattering Barriers. Nat. Mater. 2010, 9 (6), 491–495. 10.1038/nmat2752. 20436465

[ref62] Losego M. D.; Grady M. E.; Sottos N. R.; Cahill D. G.; Braun P. V. Effects of Chemical Bonding on Heat Transport across Interfaces. Nat. Mater. 2012, 11 (6), 502–506. 10.1038/nmat3303. 22522593

[ref63] Hung Anh L. D.; Pásztory Z. An Overview of Factors Influencing Thermal Conductivity of Building Insulation Materials. J. Build. Eng. 2021, 44, 102604 10.1016/j.jobe.2021.102604.

[ref64] Abu-Jdayil B.; Mourad A. H.; Hittini W.; Hassan M.; Hameedi S. Traditional, State-of-the-Art and Renewable Thermal Building Insulation Materials: An Overview. Constr. Build. Mater. 2019, 214, 709–735. 10.1016/j.conbuildmat.2019.04.102.

[ref65] Patil A. O.; Ikenoue Y.; Wudl F.; Heeger A. J. Water-Soluble Conducting Polymers. J. Am. Chem. Soc. 1987, 109, 1858–1859. 10.1021/ja00240a044.

[ref66] Bandow S.; Rao A. M.; Williams K. A.; Thess A.; Smalley R. E.; Eklund P. C. Purification of Single-Wall Carbon Nanotubes by Microfiltration. J. Phys. Chem. B 1997, 101, 8839–8842. 10.1021/jp972026r.

[ref67] Quinson J.; Kunz S.; Arenz M. Surfactant-Free Colloidal Syntheses of Precious Metal Nanoparticles for Improved Catalysts. ACS Catal. 2023, 13, 4903–4937. 10.1021/acscatal.2c05998.

[ref68] Kim S. Y.; Hong J.; Kavian R.; Lee S. W.; Hyder M. N.; Shao-Horn Y.; Hammond P. T. Rapid Fabrication of Thick Spray-Layer-by-Layer Carbon Nanotube Electrodes for High Power and Energy Devices. Energy Environ. Sci. 2013, 6, 888–897. 10.1039/c2ee23318e.

[ref69] Ji Q.; Honma I.; Paek S. M.; Akada M.; Hill J. P.; Vinu A.; Ariga K. Layer-by-Layer Films of Graphene and Ionic Liquids for Highly Selective Gas Sensing. Angew. Chem., Int. Ed. 2010, 49 (50), 9737–9739. 10.1002/anie.201004929. 21077075

[ref70] Losch P.; Huang W.; Goodman E. D.; Wrasman C. J.; Holm A.; Riscoe A. R.; Schwalbe J. A.; Cargnello M. Colloidal Nanocrystals for Heterogeneous Catalysis. Nano Today 2019, 24, 15–47. 10.1016/j.nantod.2018.12.002.

