# Supplementary material for: Hierarchical Incorporation of Reduced Graphene Oxide into Anisotropic Cellulose Nanofiber Foams Improves Their Thermal Insulation
Source: ACS Appl Mater Interfaces. 2024 Aug 13;16(34):45337–46. doi: 10.1021/acsami.4c09654 (PMC11367577; doi:10.1021/acsami.4c09654)
Supplement: Supplementary file 1 — am4c09654_si_001.pdf [file am4c09654_si_001.pdf]

Hierarchical Incorporation of Reduced Graphene Oxide into Anisotropic Cellulose Nanofiber Foams Improves their Thermal Insulation

Seyed Ehsan Hadi,[† a,b] Elias Möller,[† a,c] Sina Nolte,a,[d] Agnes Åhl,[a] Olivier Donzel-Gargand,[e] Lennart Bergström,[a,b] Alexander Holm[a,b,f,*]

[a] Department of Materials and Environmental Chemistry, Stockholm University, 106 91 Stockholm, Sweden.
[b] Wallenberg Wood Science Center, Department of Materials and Environmental Chemistry, Stockholm University, Stockholm, 10691, Sweden
[c] Department of Chemistry, Philipps-Universität Marburg, 35032 Marburg, Germany.
[d] Institute of Inorganic Chemistry, Leibniz University Hannover, D-30167 Hannover, Germany
[e] Ångström Solar Center, Division of Solar Cell Technology, Uppsala University, 751 21 Uppsala, Sweden
[f] Laboratory of Organic Electronics, Department of Science and Technology (ITN), Linköping University, Norrköping, SE-60174 Sweden

[†] indicating equal contribution

Corresponding author
* E-mail: alexander.holm@liu.se

Table of Contents

Section S1. Miscellaneous Figures and Tables……………………………………………...page S-2

Section S2. GO and GO+ AFM image statistics………………………………...…page S-14

Section S3. Statistical significance of difference in thermal conductivity (axial and radial) between rGO – CNF foams and CNF control foams……..…………………………… page S-16

Section S4. References...page S-19

Section S1. Miscellaneous Figures and Tables

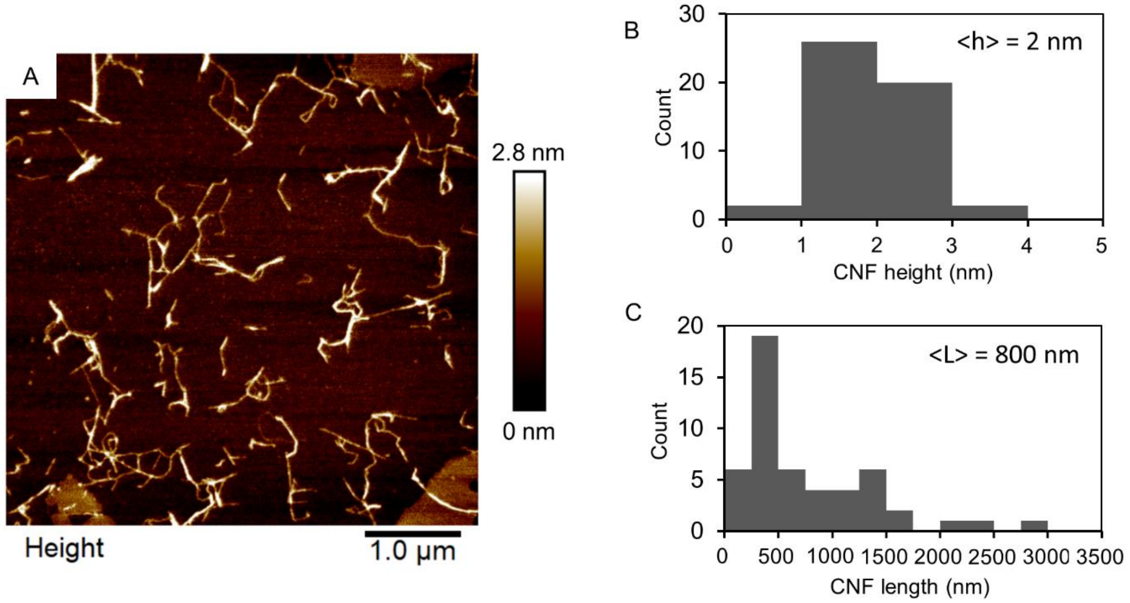

Figure S1. CNF Characterization. **(A)** AFM micrograph of CNF on freshly cleaved mica. **(B, C)** Image statistics (from AFM micrographs). **(B)** Distribution of CNF contour heights (average height, <h> = 2 nm). **(C)** Distribution of CNF contour lengths (average length 800 nm). N = 50 individual fibers were used for image statistics. Fibers that appeared entangled with other fibers were not used for length statistics. The length statistics should therefore be regarded as approximate.

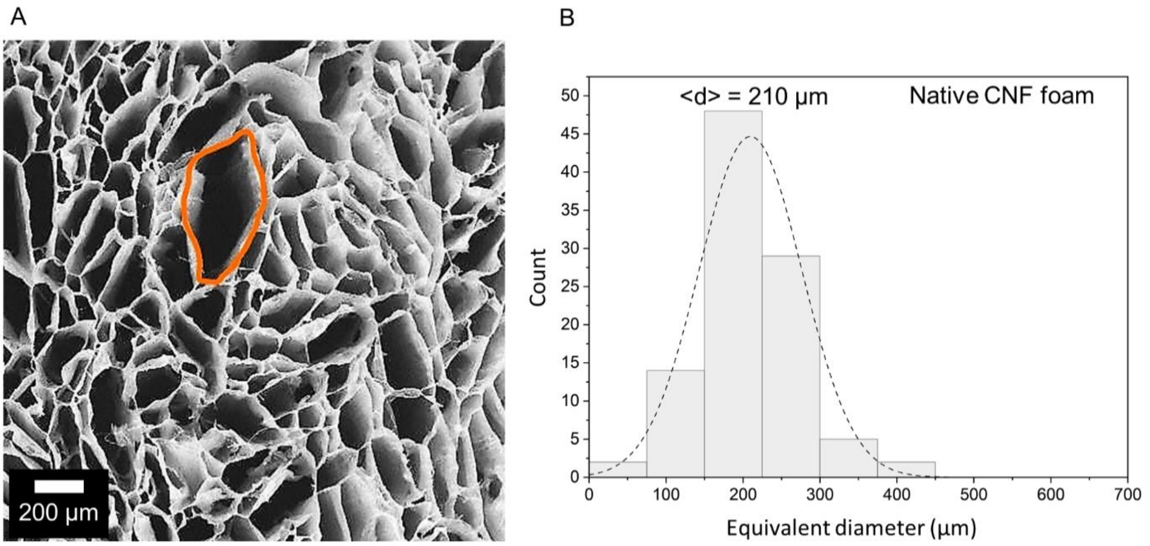

Figure S2. Image statistics to determine macropore size in native, anisotropic CNF foams. **(A)** Top view of foam. To measure macropore cell-diameters, the projected area (one cell-area marked orange for illustration) of a macropore was measured, then the equivalent disc-diameter was calculated. **(B)** Distribution of equivalent disc-diameters for native CNF foams. For the distribution, 100 individual cells were measured.

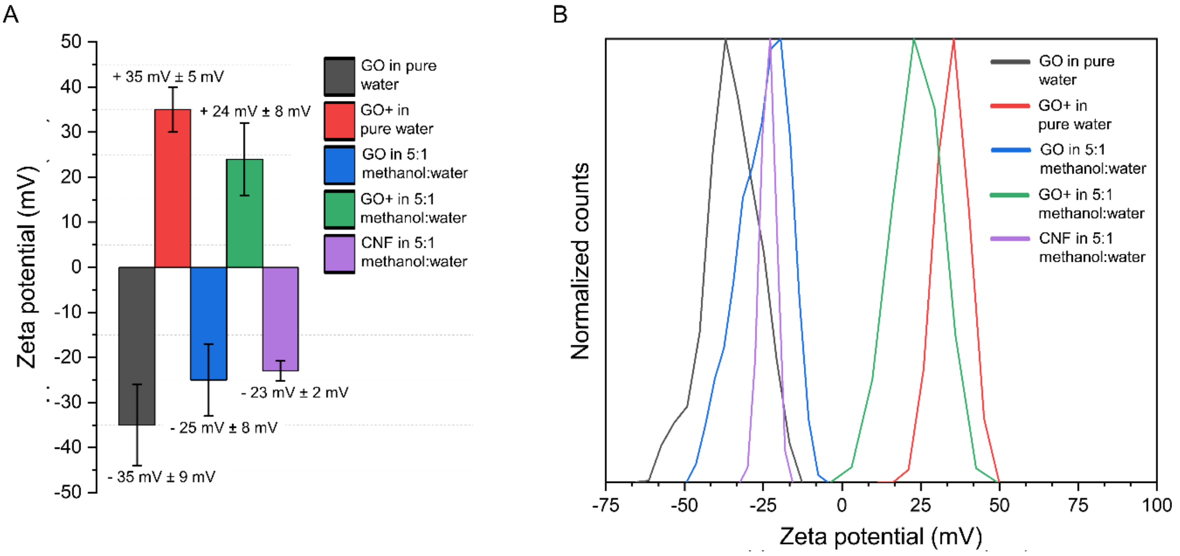

Figure S3. Analysis of zeta potentials (ζ) of: GO and GO+ in pure water and in 5:1 methanol:water mixtures, and of CNF in a 5:1 methanol:water mixture. **(A)** Illustrated by graphical charts. **(B)** Represented by zeta potential distribution curves.

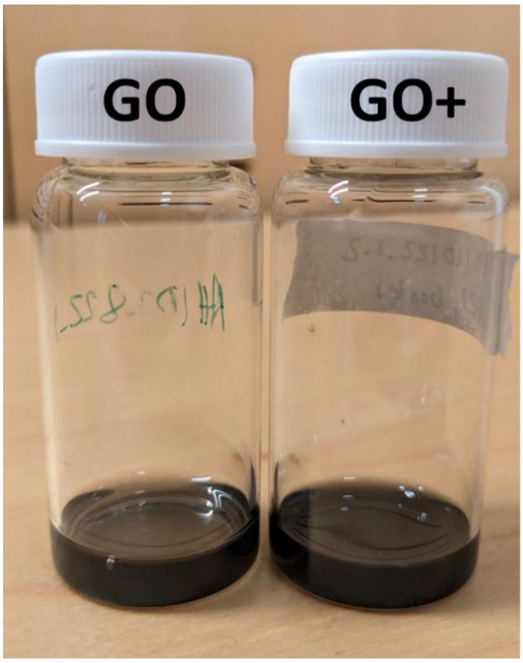

Figure S4. Dispersions of GO and GO+ in pure water after 6 months of storage.

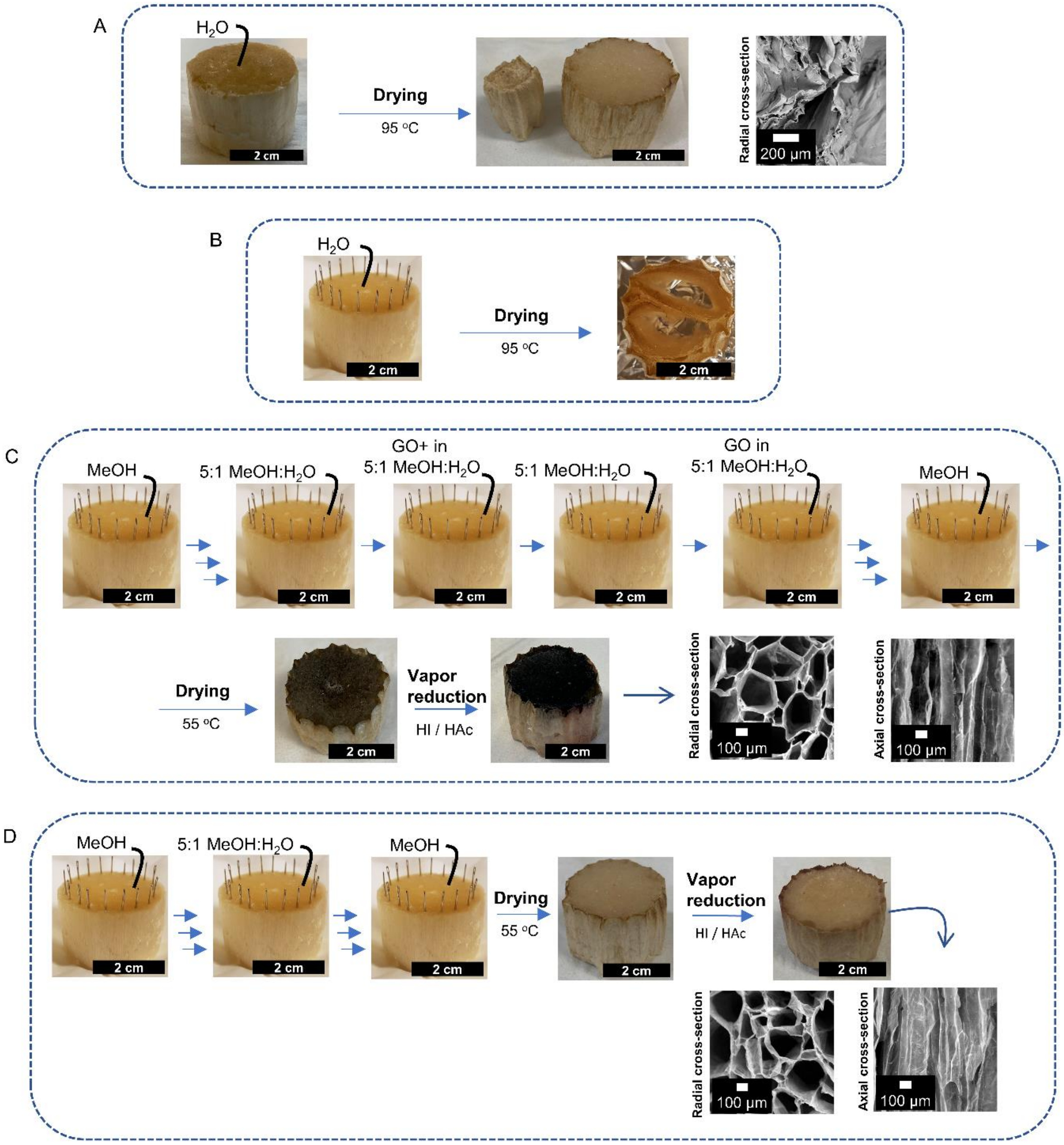

Figure S5. (A) Dropwise water-addition to the top of native CNF foam followed by drying at 95 ˚C causes the foam to contract. To the right is an image of a contracted foam, compared to a non-contracted foam that was wet-impregnated according to (D). To the right is also a top view SEM micrograph of the collapsed foam. **(B)** Dropwise water-addition to the top of affixed (with needles) native CNF foam. Upon drying (at 95 ˚C) this foam cracked (photo of cracked foam to the right). **(C)** Production of rGO – CNF foams. A foam was affixed to a Styrofoam plate using needles. The foam was then soaked by dropwise addition from the top, first with methanol, and then with methanol:water mixtures of successively higher water fractions (Table S2). After soaking the foam in 5:1 methanol:water, GO+ in 5:1 methanol:water was added to the foam, followed by GO (in 5:1 methanol:water). The foams were then soaked with methanol:water mixtures of successively lower water content, until the foams were completely soaked in pure methanol (Table S2). The foams were then dried at 55 ˚C. Finally, the foams were vapor-reduced by hydroiodic acid / acetic acid. **(D)** CNF control foams were produced exactly as described in (C) (same methanol:water compositions, same added volumes, same drying and reduction protocols), except that GO+ and GO were not added. One batch of native CNF foams were used to make both rGO – CNF foams and CNF control foams.

Table S1. Comparison of physical properties between rGO – CNF and CNF control foams. Errors are given with 95 % confidence. Refer to the methods section, main paper, for determination of densities, overall porosities, and specific heat capacities ($C_{p,dry}$). Refer to Figure S7 and S8 for determination of average macropore sizes. Refer to Figure S9 and Figure S10 for determination of number density of macropores. Refer to Figure S11 and Figure S12 for determination of BET surface areas.

	**Density (dry foams) (kg m^{-3})**	**Overall porosity (dry foams) (%)**	**Number density of macropores (mm^{-2})**	**$C_{p,dry}$ (J kg^{-1} K^{-1})**	**BET surface area (m^2 g^{-1})**	**Average macropore size (µm)**
**rGO – CNF foam**	10.0 ± 1.4	99.3 ± 0.1	18.5 ± 5.0	885 ± 23	4.99 ± 0.03	264
**CNF control foam**	10.0 ± 0.9	99.3 ± 0.1	19.9 ± 6.8	885 ± 46	4.77 ± 0.05	256

Table S2. Mixtures used in each impregnation step to make rGO – CNF foams. Note that the mixtures were chosen so that the change in surface tension was the same between each step ($\Delta\gamma \approx 0.8$ mN m^{-1}). We did this to avoid large surface tension gradients in the foams during impregnation, since we surmised that large surface tension gradients potentially could deform the foams. Surface tensions of methanol:water mixtures were taken from reference[1].

**Addition #**	**Volume (mL)**	**Mixture ($V_{methanol}$: V_{water})**	**Surface tension at 20 °C (mN m^{-1})**
1	10	Methanol	23.0
2	3	20:1 Methanol:H_2O	23.8
3	3	10:1 Methanol:H_2O	24.5
4	3	6.6:1 Methanol:H_2O	25.3
5	3	5:1 Methanol:H_2O	26.0
6	15	GO+ in 5:1 Methanol:H_2O	26.0
7	10	5:1 Methanol:H_2O	26.0
8	15	GO in 5:1 Methanol:H_2O	26.0
9	3	5:1 Methanol:H_2O	26.0
10	3	6.6:1 Methanol:H_2O	25.3
11	3	10:1 Methanol:H_2O	24.5
12	3	20:1 Methanol:H_2O	23.8
13	15	Methanol	23.0
Total volume added	89 mL		

Table S3. Mixtures used in each impregnation step to make CNF control foams. Note that the mixtures were chosen so that the change in surface tension was the same between each step ($\Delta\gamma \approx 0.8$ mN m^{-1}). The order, volume, and methanol:water composition of each addition is the exact same as for production of rGO – CNF foams (Table S2), except that GO+ and GO were not added. Surface tensions of methanol:water mixtures were taken from reference[1].

**Addition #**	**Volume (mL)**	**Mixture ($V_{methanol}$: V_{water})**	**Surface tension at 20 ˚C (mN m^{-1})**
1	10	Methanol	23.0
2	3	20:1 Methanol:H_2O	23.8
3	3	10:1 Methanol:H_2O	24.5
4	3	6.6:1 Methanol:H_2O	25.3
5	3	5:1 Methanol:H_2O	26.0
6	15	5:1 Methanol:H_2O	26.0
7	10	5:1 Methanol:H_2O	26.0
8	15	5:1 Methanol:H_2O	26.0
9	3	5:1 Methanol:H_2O	26.0
10	3	6.6:1 Methanol:H_2O	25.3
11	3	10:1 Methanol:H_2O	24.5
12	3	20:1 Methanol:H_2O	23.8
13	15	Methanol	23.0
Total volume added	89 mL		

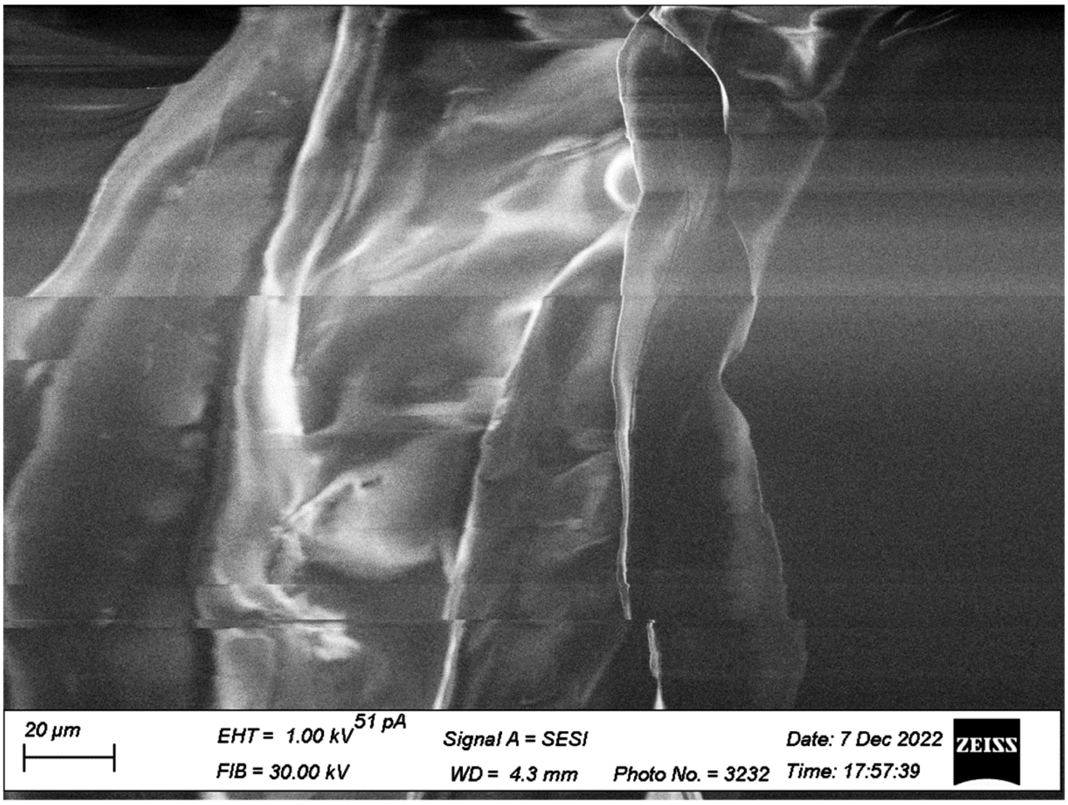

Figure S6. Typical FIB micrograph of CNF control foam. No film of sheet-like structures was observed (as in rGO – CNF foams, Figure 4 E). Imaging was difficult, because of significant charging under the beam, deforming the foam within seconds. In contrast, the rGO – CNF foams were not charging under the beam (Figure 4 E), using the same FIB settings.

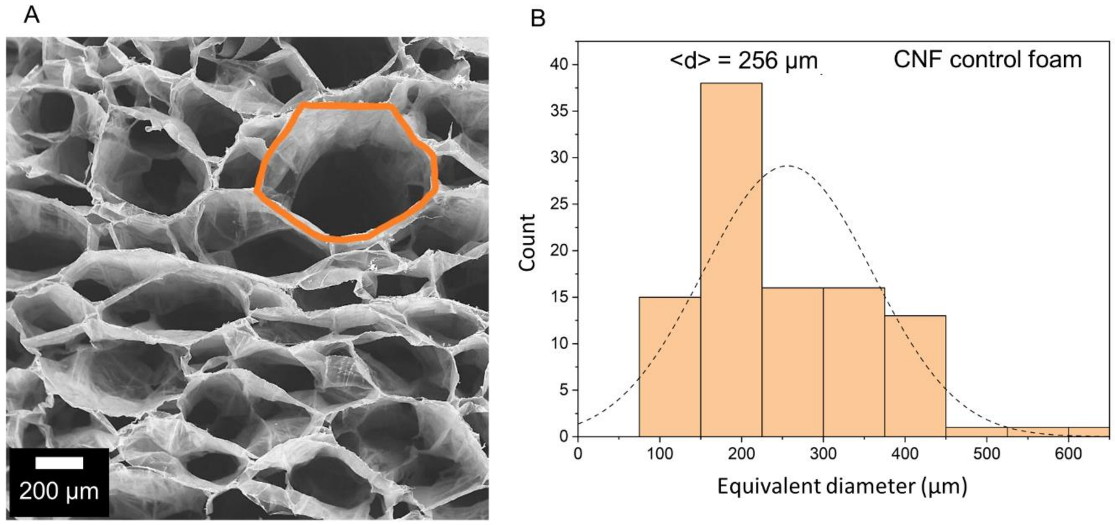

Figure S7. Image statistics to determine macropore size in CNF control foams. **(A)** Top view of foam. To measure macropore cell-diameters, the projected area (one cell-area marked orange for illustration) of a macropore was measured, then the equivalent disc-diameter was calculated. **(B)** Distribution of equivalent disc-diameters for CNF control foams. For the distribution, 100 individual cells were measured.

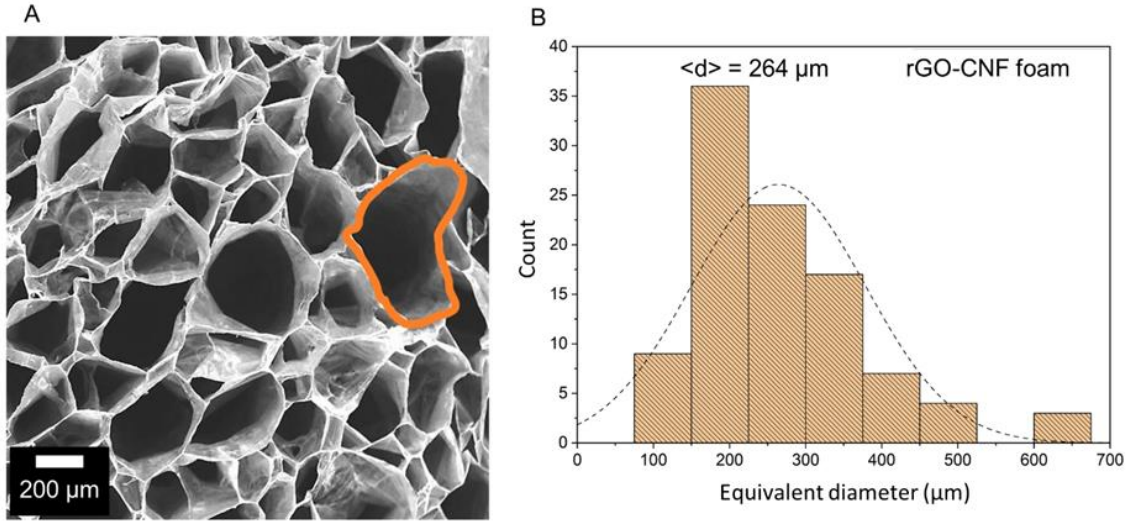

Figure S8. Image statistics to determine macropore size in rGO – CNF foams. **(A)** Top view of foam. To measure macropore cell-diameters, the projected area (one cell-area marked orange for illustration) of a macropore was measured, then the equivalent disc-diameter was calculated. **(B)** Distribution of equivalent disc-diameters for rGO – CNF foams. For the distribution, 100 individual cells were measured.

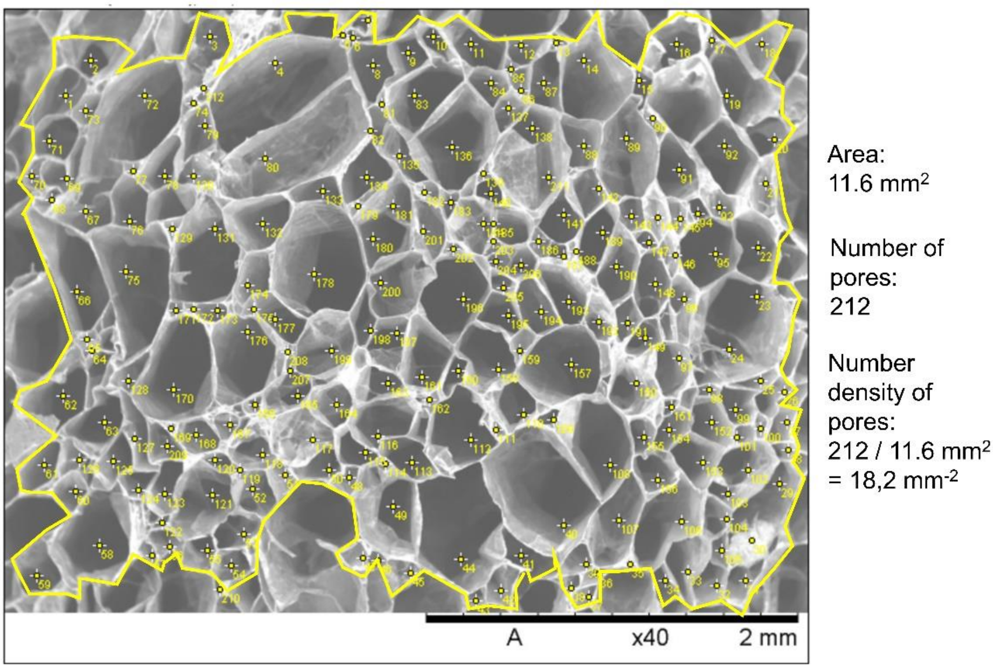

Figure S9. Illustration of how number density of macropores in rGO – CNF foams was estimated from SEM micrographs. Top view of foam. To measure number-density of macropores, all macropores wholly inside one SEM micrograph was counted, and their total area measured. The number density was then calculated as number of macropores / total cross-sectional area of macropores. 4 micrographs of different foam-regions were used, which resulted in an estimation of the number density of pores of 18.5 mm^{-2} ± 5.0 mm^{-2} (95 % confidence).

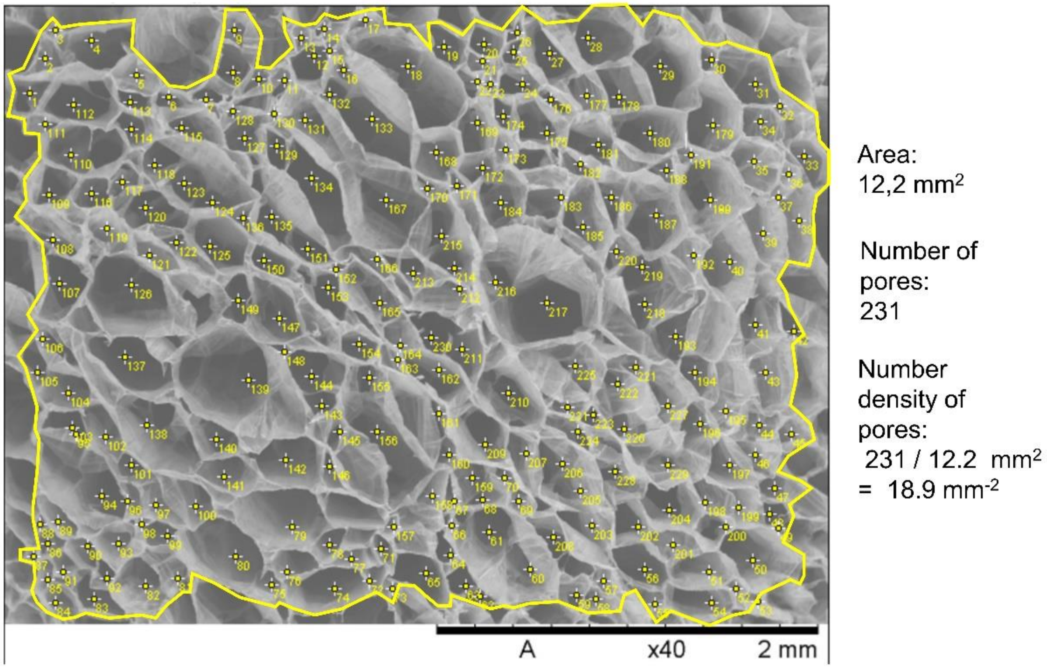

Figure S10. Illustration of how number density of macropores in CNF control foams was estimated from SEM micrographs. Top view of foam. To measure number-density of macropores, all macropores wholly inside one SEM micrograph was counted, and their total area measured. The number density was then calculated as number of macropores / total cross-sectional area of macropores. 4 micrographs of different foam-regions were used, which resulted in an estimation of the number density of pores of 19.9 mm^{-2} ± 6.8 mm^{-2} (95 % confidence).

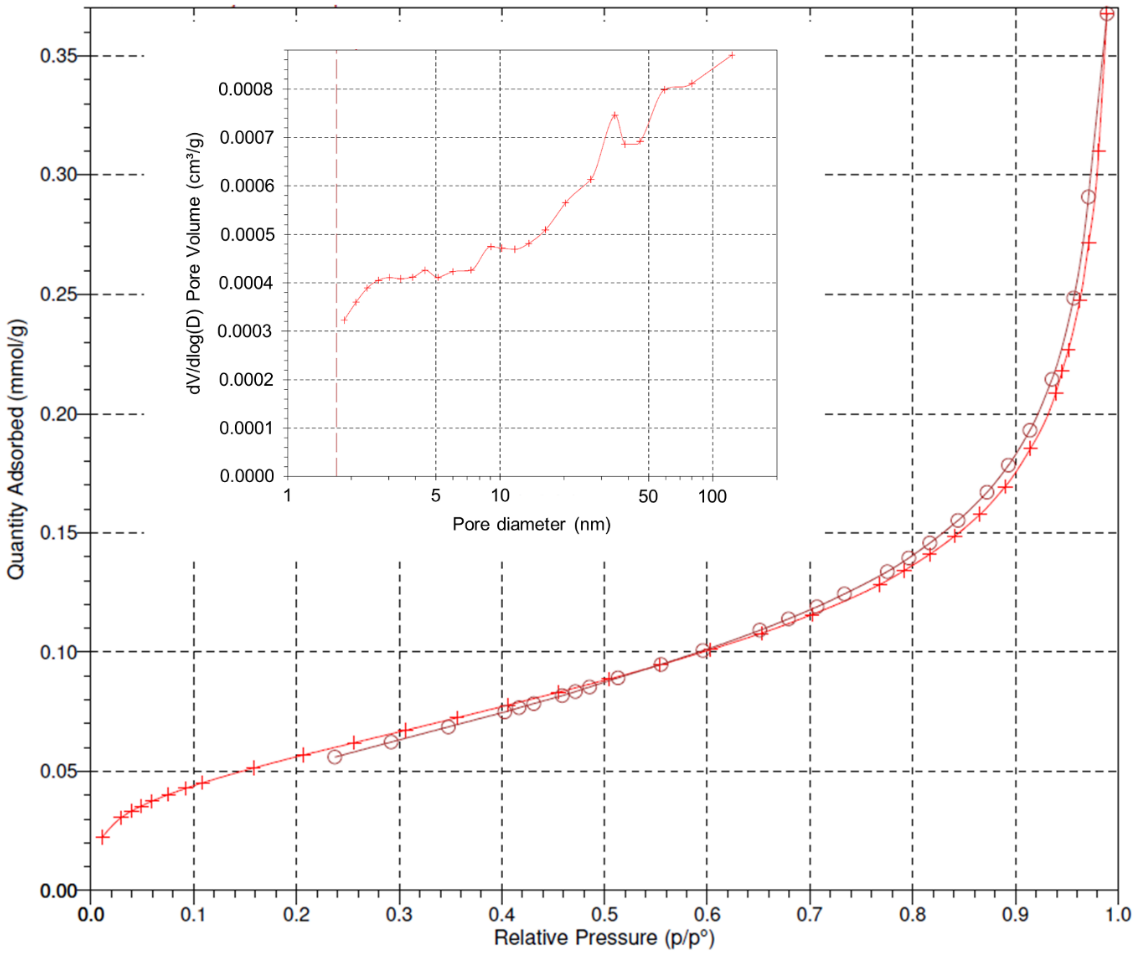

Figure S11. N_2 adsorption (crosses) and desorption (open circles) isotherms for the CNF control foams. BET analysis of the data yielded a surface area of 4.8 m^2 g^{-1}. The pore-size distribution was obtained by BJH analysis of the isotherms, and is presented in the inset. Note the similarity of the pore-size distribution between CNF control foams (here) and the rGO – CNF foams (Figure S12).

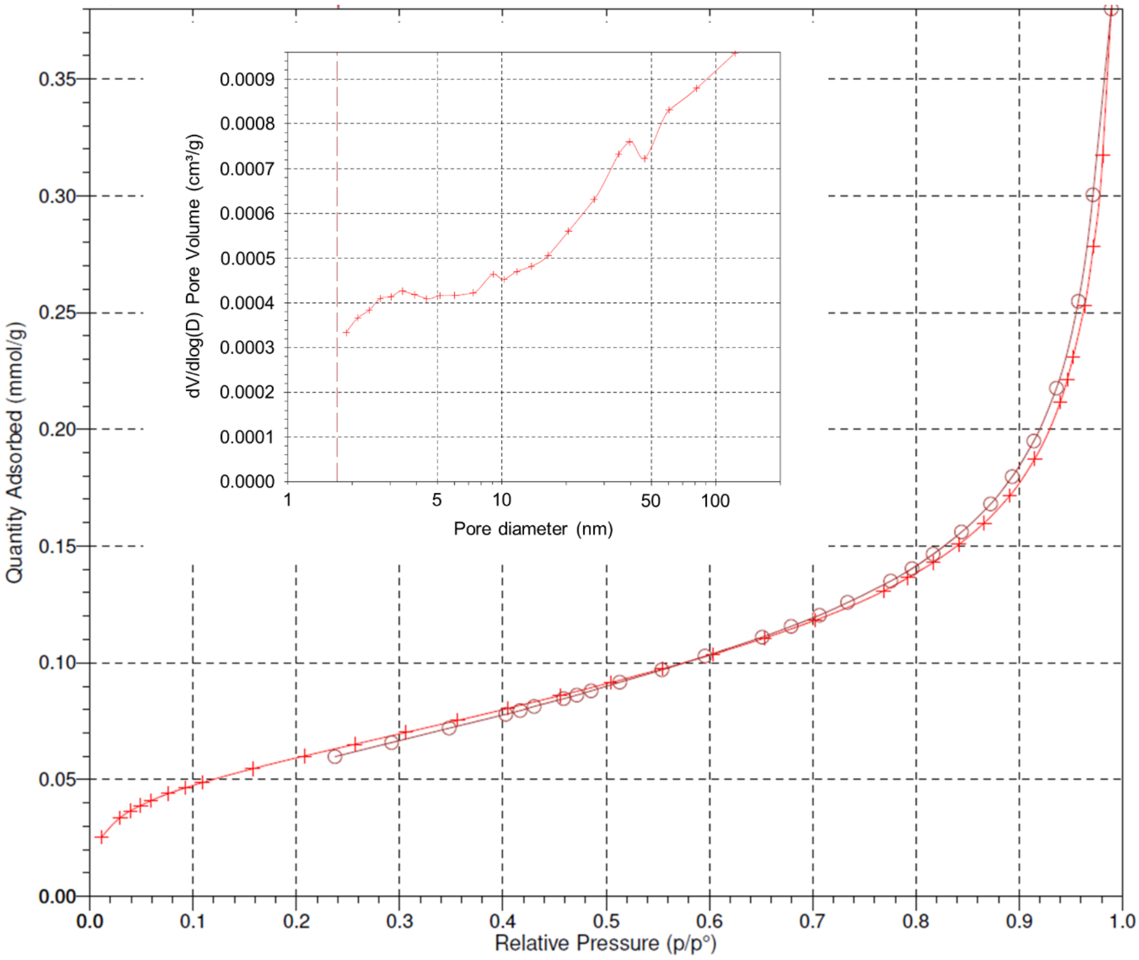

Figure S12. N_2 adsorption (crosses) and desorption (open circles) isotherms for the rGO – CNF foams. BET analysis of the data yielded a surface area of 5.0 m^2 g^{-1}. The pore-size distribution was obtained by BJH analysis of the isotherms, and is presented in the inset. Note the similarity of the pore-size distribution between the rGO – CNF foams (here) and the CNF control foams (Figure S11).

Table S4. Comparative analysis of thermal conductivity and thermal conductivity anisotropy ratio in various materials reported in the literature.

**Name of the sample**	**Density ($g\ cm^{-3}$)**	**Axial thermal conductivity ($W\ m^{-1}\ K^{-1}$)**	**Radial thermal conductivity ($W/m^{-1}\ K^{-1}$)**	**Anisotropy ratio (λ_a/ λ_r)**	**Ref**
Nanowood	0.13	0.06	0.03	2.0	2
Paraffin wax and a carbon framework composite	0.933	0.77	0.58	1.3	3
Polyurethane and carbon nanotubes composite	N/A	@T=25°C: 2.3	@T=25°C: 0.9	2.5	4
p-aramid nanofibers aerogel	0.025	@T=25°C: 0.037	@T=25°C: 0.041	0.9	5
Cyclotriphosphazene-bridged periodic mesoporous organosilica /CNF foam	0.0166	0.049	0.027	1.8	6
rGO/polyimide nanocomposite foam	0.008	0.038	0.012	3.8	7
Anisotropic CNF/Silica foam	0.020	@RH=50%: 0.112	@RH=50%: 0.030	3.7	8
Anisotropic polyimide/bacterial cellulose aerogel	0.046	0.066	0.030	2.2	9
Anisotropic CNF/chitosan aerogel	0.0134	0.032	0.026	1.2	10
Hydroxyapatite nanowires/polyimide aerogel	0.0431	@T=25°C 0.038	@T=25°C 0.036	1.0	11
Halloysite nanotubes/gelatin-based aerogel	0.0575	0.040	0.037	1.1	12
CNF/methylcellulose/tannic acid foams	0.018	@RH=50%: 0.115	@RH=50%: 0.023	5	13
Carbon/graphene composite aerogels	0.069	0.196	0.038	5.1	14
Polyimide/SiO_2 composite aerogels	0.1302	0.048	0.028	1.7	15
AgNWs/carbon aerogels	0.008	@RH=50%: 0.090	@RH=50%: 0.019	4.7	16
SiO_2/CNF aerogels	0.247	@T=25°C: 0.035	@T=25°C: 0.017	2.1	17
Chitosan-based/epoxy thermoset aerogels	0.057	0.041	0.035	1.2	18
Unidirectionally freeze-cast CNF foam	0.064	0.15	0.018	8	19
Unidirectionally freeze-cast CNF / graphene oxide / baric acid / sepiolite foam	0.075	0.17	0.015	11	20

Uni-directionally freeze-cast CNF-bPEI-Tannic acid	0.077	0.10	0.026	3.8	[21]
**rGO-CNF foam**	**0.0110**	**@RH=50%: 0.076**	**@RH=50%: 0.030**	**2.5**	**This work**
**CNF Control foam**	**0.0111**	**@RH=50%: 0.078**	**@RH=50%: 0.033**	**2.4**	**This work**

Table S5. Electrical conductivity, in the axial direction, of the rGO-CNF foam, and the CNF control foam.

Sample	Electrical conductivity (µS cm^{-1})
rGO-CNF foam	2600 ± 400
CNF Control foam	Below detection limit of instrument

Table S6. Relative humidity (RH) dependent densities (kg m^{-3}) of rGO – CNF foams and CNF control foams. Three foams of each type were used for measurement. Errors are given with 95 % confidence.

	Relative Humidity (RH, %)					
	**0**	**20**	**35**	**50**	**65**	**80**
**rGO – CNF foam density (kg m^{-3})**	10.0 ± 1.4	11.1 ± 1.6	11.2 ± 1.6	11.4 ± 1.6	11.6 ± 1.6	12.1 ± 1.7
**CNF control foam density (kg m^{-3})**	10.0 ± 0.9	11.0 ± 1.1	11.2 ± 1.1	11.5 ± 1.1	11.9 ± 1.2	12.7 ± 1.3

Section S2. GO and GO+ AFM image statistics

Image statistics for GO and GO+ were generated by depositing GO or GO+ suspensions in 5:1 methanol:water on a SiO_2 wafer, followed by AFM characterization. For both height and roughness statistics, 10 individual sheets were measured for both GO and GO+. An illustration of how the height of one individual sheet was measured is given in Figure S13. An illustration of how the roughness of one individual sheet was measured is given in Figure S14. For calculation of 95 % confidence intervals about the mean heights ($\overline{h_{GO}}$ and $\overline{h_{GO+}}$) and mean roughnesses ($\overline{R_{a,GO}}$ and $\overline{R_{a,GO}}$), gaussian distributions of errors were assumed.

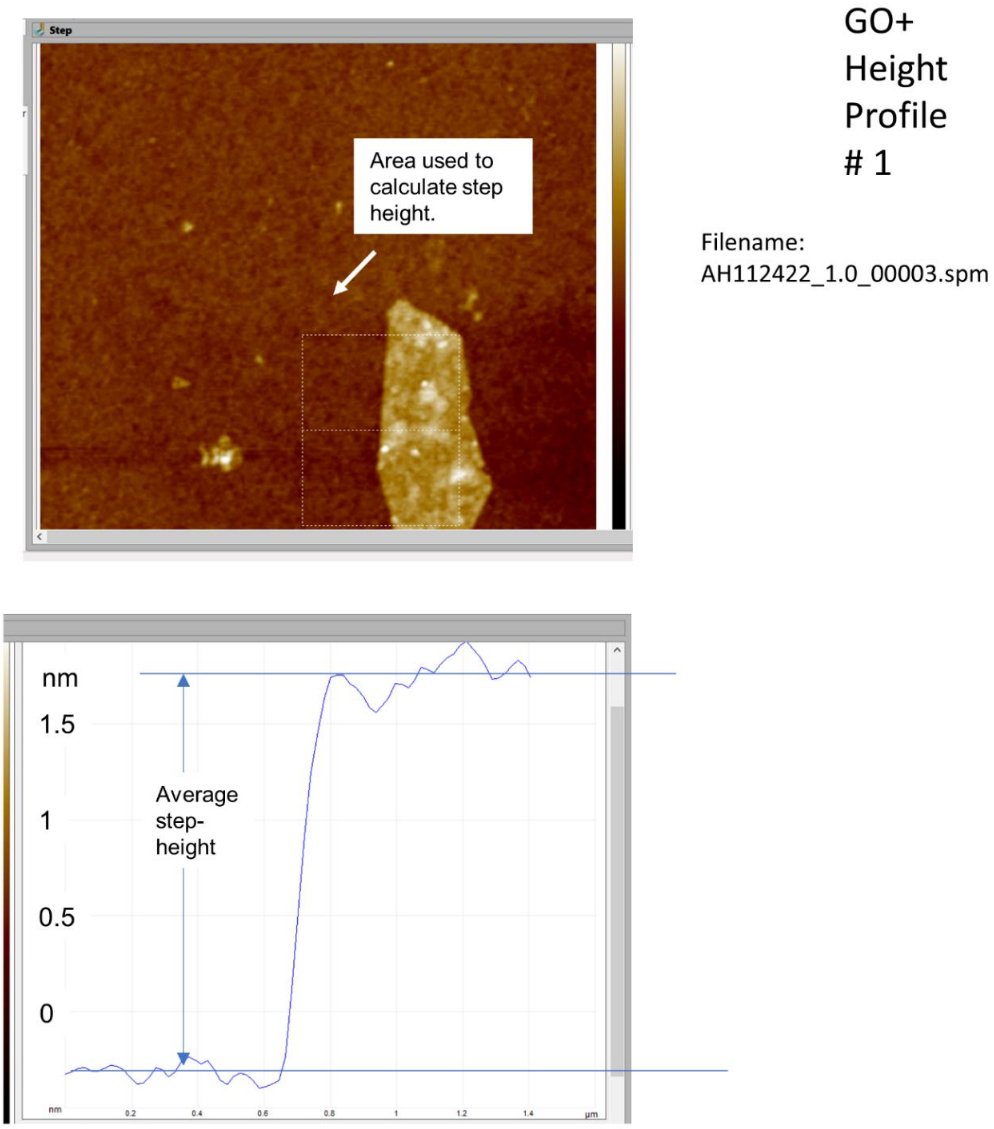

Figure S13. Illustration of how height in one individual GO+ sheet was measured. GO sheets were measured in the same fashion. 10 individual sheets were used to determine the average sheet height.

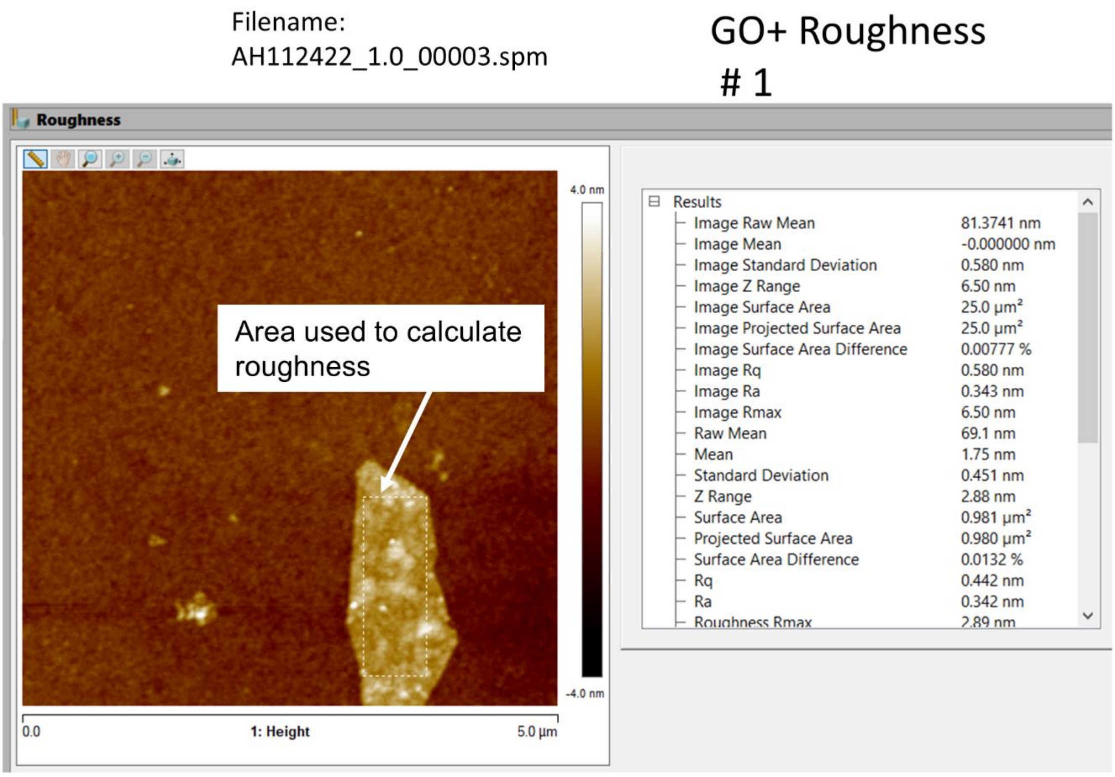

Figure S14. Illustration of how roughness (R_a) in one individual GO+ sheet was measured. GO sheets were measured in the same fashion. 10 individual sheets were used to determine the average sheet roughness.

Section S3. Statistical significance of difference in thermal conductivity (axial and radial) between rGO – CNF and CNF control foams.

t-tests to judge significance of differences between thermal conductivities at each RH. At each relative humidity, and for each type of sample (rGO – CNF foam or CNF control foam), axial and radial thermal conductivity was measured in three individually produced foam-pairs (one pair is needed for one individual thermal conductivity measurement). Below, we illustrate how, using a two-sided t-test,[22] these measurements are used to determine with which significance the radial thermal conductivity in rGO – CNF foams are lower than in CNF control foams at RH 20 %. The determinations of the respective significances at other relative humidities are analogous. At RH 20 %, we determine the following quantities:

The subscript $_1$ denotes the rGO – CNF foam, $_2$ denotes the CNF control foam.

$\overline{\lambda_{r,1}}$: Estimation of the true radial conductivity ($\lambda_{r,1}$), of the rGO – CNF foam. Given by the average of three individual measurements. At RH 20%, we have $\overline{\lambda_{r,1}} =$ 33.2 mW m^{-1} K^{-1}.

$\overline{\lambda_{r,2}}$: Estimation of the true radial conductivity ($\lambda_{r,2}$), of the CNF control foam. Given by the average of three individual measurements. At RH 20%, we have $\overline{\lambda_{r,2}} =$ 37.9 mW m^{-1} K^{-1}.

$S_{r,1}$: Estimation of standard deviation for the measurement of $\lambda_{r,1}$. At RH 20%, we have $S_{r,1} =$ 5.8 mW m^{-1} K^{-1}.

$S_{r,2}$: Estimation of standard deviation for the measurement of $\lambda_{r,2}$. At RH 20%, we have $S_{r,2} =$ 2.3 mW m^{-1} K^{-1}.

n_1 Number of individual experimental estimations of $\lambda_{r,1}$. For RH 20%, and at all other RHs, $n_1 = 3$.

n_2 Number of individual experimental estimations of $\lambda_{r,2}$. For RH 20%, and at all other RHs, $n_2 = 3$.

We then form the null hypothesis (H_0), that the true radial thermal conductivities at RH=20% are the same, i.e.

H_0: $\lambda_{r,1} = \lambda_{r,2}$

Because the radial thermal conductivities for the rGO – CNF foams and CNF control foams are similar, and because the thermal conductivities are measured on one instrument (TPS 2500 S) by the same procedure, we assume that the variances for the experimental estimations of $\lambda_{r,1}$ and $\lambda_{r,2}$ are approximately equal so that a pooled estimation of the standard deviation can be introduced.[22] At RH 20%, we have:

$$S_p = \sqrt{\frac{(n_1-1)S_{r,1}^2+(n_1-1)S_{r,2}^2}{n_1+n_2-2}} = \sqrt{\frac{(3-1)5.8^2+(3-1)2.3^2}{3+3-2}} = 4.4 \text{ mW m}^{-1} \text{ K}^{-1}.$$

The degrees of freedom (*d.f.*) for the pooled standard deviation is:

d.f. = $n_1 + n_2 - 2 = 3 + 3 - 2 = 4$

We can now introduce the statistic *t*:

$$t = \frac{\overline{\lambda_{r,2}} - \overline{\lambda_{r,2}}}{S_p\sqrt{\frac{1}{n_2}+\frac{1}{n_1}}} = \frac{37.9 - 33.2}{4.4\sqrt{\frac{1}{3}+\frac{1}{3}}} = 1.31$$

For *d.f.* = 4, this value for the statistic t, gives a p-value of 0.26. The null hypothesis (**H0:** $\lambda_{r,1} = \lambda_{r,2}$) can therefore be rejected at the 74 % level of significance. i.e., at RH 20 %, the radial thermal conductivity of rGO – CNF foams is lower than the radial thermal conductivity of CNF control foams with 74 % significance. At the remaining RHs (35, 50, 65, 80) %, the t-test gave the significances (89, 81, 99, 95) % that the radial thermal conductivity of rGO – CNF foams is lower than the radial thermal conductivity of CNF control foams.

A two-sided t-test was applied to the axial thermal conductivities in an analogous fashion as described above. The p-values for the null hypothesis **H0:** $\lambda_{a,1} = \lambda_{a,2}$ were (0.67, 0.32, 0.62, 0.96, 0.51) at RH (20, 35, 50, 65, 80) %. The null hypothesis is therefore retained at (67, 32, 62, 96, 51) % levels of significance for RH (20, 35, 50, 65, 80) %, meaning there is no experimental support that the axial thermal conductivity differs between the rGO – CNF foams and the CNF control foams at any RH.

Two-sided, paired t-test[22] to judge significance that the radial thermal conductivity overall is lower in rGO – CNF foams than in CNF control foams. At each RH, we form the difference between the estimation of radial thermal conductivity in the CNF control foam ($\overline{\lambda_{r,2}}$) and the estimation of radial thermal conductivity in the rGO – CNF foam ($\overline{\lambda_{r,1}}$). This creates a set of differences, in our case d = (4.7, 4.6, 2.8, 4.3, 8.0) mW m^{-1} K^{-1}. A null hypothesis can then be formed that the estimations $\overline{\lambda_{r,1}}$ and $\overline{\lambda_{r,2}}$ pairwise (at each RH) are drawn from the same population, or equivalently that the average of the pairwise differences (μ_d) is zero, i.e.:

H0: $\mu_d = 0$

This null hypothesis gives rise to the statistic t:[22]

$$t = \frac{\bar{d}\sqrt{n}}{S_d}$$

where $\bar{d}$ is the experimental estimation (calculated from the *d*-values obtained above) of the true average of the pairwise differences (μ_d), *n* is the number of pair-wise differences and S_d is the estimation of the standard deviation for the measurement of d. For our set of pair-wise differences, we have $\bar{d}$ = 4.9 mW m^{-1} K^{-1}, S_d = 1.9 mW m^{-1} K^{-1} and *n* = 5, giving the following t-value:

$$t = \frac{4.9\sqrt{5}}{1.9} = 5.8$$

For *d.f.* = 5 – 1 = 4, this value for the statistic t, gives a (two-sided) p-value of 0.005. Thus, the null hypothesis, **H0:** $\mu_d = 0$ can be rejected with 99.5 % significance, suggesting very strongly that, $\lambda_{r,1} < \lambda_{r,2}$ at all considered RHs. I.e., the paired t-test strongly suggests that the radial thermal conductivity of rGO – CNF foams are lower than the radial thermal conductivity in CNF control foams, irrespective of the RH.

Paired t-test to judge significance that the axial thermal conductivity overall is different in rGO – CNF foams compared to in CNF control foams. This two-sided paired t-test is carried out in analogy with the above-described paired t-test for the radial thermal conductivities. In this case we find that the p-value for the null hypothesis **H_0:** $\mu_d = 0$ is p = 0.32. The null hypothesis is therefore retained with a significance of 32 %. Therefore, considering difference in axial thermal conductivities at all RHs, there is no experimental support that the axial thermal conductivity differs between the rGO – CNF foams and the CNF control foams.

Section S4. References

(1) Vázquez, G.; Alvarez, E.; Navaza, J. M. *Surface Tension of Alcohol + Water from 20 to 50 °C*; 1995; Vol. 40. https://pubs.acs.org/sharingguidelines.

(2) Li, T.; Song, J.; Zhao, X.; Yang, Z.; Pastel, G.; Xu, S.; Jia, C.; Dai, J.; Chen, C.; Gong, A.; Jiang, F.; Yao, Y.; Fan, T.; Yang, B.; Wågberg, L.; Yang, R.; Hu, L. Anisotropic, Lightweight, Strong, and Super Thermally Insulating Nanowood with Naturally Aligned Nanocellulose. *Sci Adv* **2018**, *4*, eaar3724.

(3) Sheng, N.; Zhu, R.; Dong, K.; Nomura, T.; Zhu, C.; Aoki, Y.; Habazaki, H.; Akiyama, T. Vertically Aligned Carbon Fibers as Supporting Scaffolds for Phase Change Composites with Anisotropic Thermal Conductivity and Good Shape Stability. *J Mater Chem A Mater* **2019**, *7* (9), 4934–4940. https://doi.org/10.1039/c8ta11329g.

(4) Aftab, W.; Mahmood, A.; Guo, W.; Yousaf, M.; Tabassum, H.; Huang, X.; Liang, Z.; Cao, A.; Zou, R. Polyurethane-Based Flexible and Conductive Phase Change Composites for Energy Conversion and Storage. *Energy Storage Mater* **2019**, *20*, 401–409. https://doi.org/10.1016/j.ensm.2018.10.014.

(5) Xie, C.; He, L.; Shi, Y.; Guo, Z. X.; Qiu, T.; Tuo, X. From Monomers to a Lasagna-like Aerogel Monolith: An Assembling Strategy for Aramid Nanofibers. *ACS Nano* **2019**, *13* (7), 7811–7824. https://doi.org/10.1021/acsnano.9b01955.

(6) Wang, D.; Feng, X.; Zhang, L.; Li, M.; Liu, M.; Tian, A.; Fu, S. Cyclotriphosphazene-Bridged Periodic Mesoporous Organosilica-Integrated Cellulose Nanofiber Anisotropic Foam with Highly Flame-Retardant and Thermally Insulating Properties. *Chemical Engineering Journal* **2019**, *375*. https://doi.org/10.1016/j.cej.2019.121933.

(7) Qin, Y.; Peng, Q.; Zhu, Y.; Zhao, X.; Lin, Z.; He, X.; Li, Y. Lightweight, Mechanically Flexible and Thermally Superinsulating RGO/Polyimide Nanocomposite Foam with an Anisotropic Microstructure. *Nanoscale Adv* **2019**, *1* (12), 4895–4903. https://doi.org/10.1039/c9na00444k.

(8) Munier, P.; Apostolopoulou-Kalkavoura, V.; Persson, M.; Bergström, L. Strong Silica-Nanocellulose Anisotropic Composite Foams Combine Low Thermal Conductivity and Low Moisture Uptake. *Cellulose* **2020**, *27* (18), 10825–10836. https://doi.org/10.1007/s10570-019-02912-0.

(9) Zhang, X.; Zhao, X.; Xue, T.; Yang, F.; Fan, W.; Liu, T. Bidirectional Anisotropic Polyimide/Bacterial Cellulose Aerogels by Freeze-Drying for Super-Thermal Insulation. *Chemical Engineering Journal* **2020**, *385*. https://doi.org/10.1016/j.cej.2019.123963.

(10) Zhang, M.; Jiang, S.; Han, F.; Li, M.; Wang, N.; Liu, L. Anisotropic Cellulose Nanofiber/Chitosan Aerogel with Thermal Management and Oil Absorption Properties. *Carbohydr Polym* **2021**, *264*. https://doi.org/10.1016/j.carbpol.2021.118033.

(11) Zhu, J.; Zhao, F.; Peng, T.; Liu, H.; Xie, L.; Jiang, C. Highly Elastic and Robust Hydroxyapatite Nanowires/Polyimide Composite Aerogel with Anisotropic Structure for Thermal Insulation. *Compos B Eng* **2021**, *223*. https://doi.org/10.1016/j.compositesb.2021.109081.

(12) Zhao, F.; Liu, H.; Li, H.; Cao, Y.; Hua, X.; Ge, S.; He, Y.; Jiang, C.; He, D. Cogel Strategy for the Preparation of a “Thorn”-Like Porous Halloysite/Gelatin Composite Aerogel with Excellent

Mechanical Properties and Thermal Insulation. *ACS Appl Mater Interfaces* **2022**, *14* (15), 17763–17773. https://doi.org/10.1021/acsami.1c23647.

(13) Church, T. L.; Kriechbaum, K.; Schiele, C.; Apostolopoulou-Kalkavoura, V.; Hadi, S. E.; Bergström, L. A Stiff, Tough, and Thermally Insulating Air- and Ice-Templated Plant-Based Foam. *Biomacromolecules* **2022**, *23* (6), 2595–2602. https://doi.org/10.1021/acs.biomac.2c00313.

(14) Jiang, X.; Zhao, Z.; Zhou, S.; Zou, H.; Liu, P. Anisotropic and Lightweight Carbon/Graphene Composite Aerogels for Efficient Thermal Insulation and Electromagnetic Interference Shielding. *ACS Appl Mater Interfaces* **2022**, *14* (40), 45844–45852. https://doi.org/10.1021/acsami.2c13000.

(15) Shao, H.; Fei, Z.; Li, X.; Zhang, Z.; Zhao, S.; Li, K.; Yang, Z. Polyimide/SiO2 Composite Aerogels with Excellent Thermal and Sound Insulation Properties Prepared by Confined Filling Method. *Mater Lett* **2024**, *354*. https://doi.org/10.1016/j.matlet.2023.135402.

(16) Zhang, M.; Fan, Y.; Wang, N.; Gao, H.; Zhang, L.; Zhao, Y.; Liu, L. Silver Nanowire-Infused Carbon Aerogel: A Multifunctional Nanocellulose-Derived Material for Personal Thermal Management. *Carbohydr Polym* **2024**, *324*. https://doi.org/10.1016/j.carbpol.2023.121470.

(17) Long, X.; Wei, X.; Hu, M.; Yu, J.; Wang, S.; Zhou, L.; Liao, J. Anisotropic and High-Strength SiO2/Cellulose Nanofiber Composite Aerogel with Thermal Superinsulation and Superhydrophobicity. *Ceram Int* **2023**, *49* (17), 28621–28628. https://doi.org/10.1016/j.ceramint.2023.06.116.

(18) Zhang, C.; Song, S.; Cao, Q.; Li, J.; Liu, Q.; Zhang, S.; Jian, X.; Weng, Z. Improving the Comprehensive Properties of Chitosan-Based Thermal Insulation Aerogels by Introducing a Biobased Epoxy Thermoset to Form an Anisotropic Honeycomb-Layered Structure. *Int J Biol Macromol* **2023**, *246*. https://doi.org/10.1016/j.ijbiomac.2023.125616.

(19) Apostolopoulou-Kalkavoura, V.; Hu, S.; Lavoine, N.; Garg, M.; Linares, M.; Munier, P.; Zozoulenko, I.; Shiomi, J.; Bergström, L. Humidity-Dependent Thermal Boundary Conductance Controls Heat Transport of Super-Insulating Nanofibrillar Foams. *Matter* **2021**, *4* (1), 276–289. https://doi.org/10.1016/j.matt.2020.11.007.

(20) Wicklein, B.; Kocjan, A.; Salazar-Alvarez, G.; Carosio, F.; Camino, G.; Antonietti, M.; Bergström, L. Thermally Insulating and Fire-Retardant Lightweight Anisotropic Foams Based on Nanocellulose and Graphene Oxide. *Nat Nanotechnol* **2015**, *10*, 277–283. https://doi.org/10.1038/nnano.2014.248.

(21) Kriechbaum, K.; Apostolopoulou-Kalkavoura, V.; Munier, P.; Bergström, L. Sclerotization-Inspired Aminoquinone Cross-Linking of Thermally Insulating and Moisture-Resilient Biobased Foams. *ACS Sustain Chem Eng* **2020**, *8* (47), 17408–17416. https://doi.org/10.1021/acssuschemeng.0c05601.

(22) Miller, J. N.; Miller, J. C. *Statistics and Chemometrics for Analytical Chemistry*, 5th ed.; Pearson: Harlow, 2005.
